# Association and interaction of *PPAR*-complex gene variants with latent traits of left ventricular diastolic function

**DOI:** 10.1186/1471-2350-11-65

**Published:** 2010-04-28

**Authors:** Jyh-Ming Jimmy Juang, Lisa de las Fuentes, Alan D Waggoner, C Charles Gu, Víctor G Dávila-Román

**Affiliations:** 1 Cardiovascular Imaging and Clinical Research Core Laboratory, Cardiovascular Division, Department of Medicine, Washington University School of Medicine, St. Louis, Missouri, USA; 2 Division of Biostatistics, Washington University School of Medicine, St. Louis, Missouri, USA; 3 Department of Genetics, Washington University School of Medicine, St. Louis, Missouri, USA; 4 Division of Cardiology, Department of Internal Medicine, National Taiwan University Hospital and National Taiwan University College of Medicine, Taipei, Taiwan

## Abstract

**Background:**

Abnormalities in myocardial metabolism and/or regulatory genes have been implicated in left ventricular systolic dysfunction. However, the extent to which these modulate left ventricular diastolic function (LVDF) is uncertain.

**Methods:**

Independent component analysis was applied to extract latent LVDF traits from 14 measured echocardiography-derived endophenotypes of LVDF in 403 Caucasians. Genetic association was assessed between measured and latent LVDF traits and 64 single nucleotide polymorphisms (SNPs) in three peroxisome proliferator-activated receptor *(PPAR)*-complex genes involved in the transcriptional regulation of fatty acid metabolism.

**Results:**

By linear regression analysis, 7 SNPs (4 in *PPARA*, 2 in *PPARGC1A*, 1 in *PPARG*) were significantly associated with the latent LVDF trait, whereas a range of 0-4 SNPs were associated with each of the 14 measured echocardiography-derived endophenotypes. Frequency distribution of *P *values showed a greater proportion of significant associations with the latent LVDF trait than for the measured endophenotypes, suggesting that analyses of the latent trait improved detection of the genetic underpinnings of LVDF. Ridge regression was applied to investigate within-gene and gene-gene interactions. In the within-gene analysis, there were five significant pair-wise interactions in *PPARGC1A *and none in *PPARA *or *PPARG*. In the gene-gene analysis, significant interactions were found between rs4253655 in *PPARA *and rs1873532 (p = 0.02) and rs7672915 (p = 0.02), both in *PPARGC1A*, and between rs1151996 in *PPARG *and rs4697046 in *PPARGC1A *(p = 0.01).

**Conclusions:**

Myocardial metabolism *PPAR*-complex genes, including within and between genes interactions, may play an important role modulating left ventricular diastolic function.

## Background

Both animal models of left ventricular (LV) pressure-overload and clinical studies in humans with hypertension implicate abnormal myocardial metabolism in the development of hypertensive heart disease (HHD) which is characterized by phenotypes such as LV hypertrophy (LVH), left ventricular diastolic dysfunction (LVDD), left ventricular systolic dysfunction (LVSD), and/or the development of heart failure (HF). Myocardial fatty acid metabolism is a key modulator of HHD phenotypes and peroxisome proliferator-activated receptor *(PPAR)*-complex genes play a critical role in regulating these metabolic processes [1-3].

The assessment of LV diastolic function (LVDF) by Doppler echocardiography is challenging due to the age-dependency of measurements, the confounding effect of LVH, and the non-linear distribution of many LVDF parameters such that no single measurement has emerged that defines LVDD [4-6]. As LVDD lies on a continuum between normal LVDF and diastolic heart failure (DHF), it represents a relevant clinical diagnosis for several reasons. LVDD, in the presence of normal LV systolic function, occurs in approximately 25% of adults 45 years of age or older and is a multivariate predictor of all-cause mortality after controlling for age, sex, and LVSD (hazards ratio >8)[6]. The most severe form of LVDD, DHF, accounts for 40-50% of the approximately 5.7 million patients with HF in the US[7].

We have previously shown that independent component analysis (ICA)-derived latent traits of HHD can be used to enhance the detection of genetic association[8]. Herein we apply ICA to derive a single latent LVDF trait from multiple echocardiography-derived indices (or "endophenotypes") of LVDF. We tested the hypothesis that *PPAR*-complex gene variants modulate LVDF traits by examining individual SNP associations and within-gene and gene-gene interaction effects.

## Methods

### I. Study population

Caucasian adults (n = 403) were analyzed from among a multi-racial cohort of consecutive subjects (n = 543) genotyped as part of a prospective genotype-phenotype association study of hypertensive heart disease at Washington University. Subjects exhibited a wide-range of cardiovascular and/or metabolic phenotypes, and also included healthy volunteers. Exclusion criteria included: 1) Hispanic ethnicity; 2) incomplete echocardiogram; 3) LVH due to conditions other than hypertension (i.e, hypertrophic cardiomyopathy); 4) significant valvular heart disease (regurgitation and/or stenosis > mild); or 5) significant systemic disease (i.e. malignancy, creatinine ≥0.22 mmol/dL [2.5 mg/dL]).

Subject demographics were assessed by interview. Heart rate, blood pressure, and anthropomorphic measurements were obtained according to a standard protocol[9]. All subjects had blood drawn for fasting glucose, insulin, and lipids; DNA was extracted from peripheral blood leukocytes. The study was approved by the Human Research Protection Office at Washington University; all subjects provided written informed consent.

### II. Echocardiography

The echocardiographic study was performed with an ultrasound system (Acuson-Siemens Sequoia, Mountain View, CA) using a 3.5-MHz array transducer. LV end-systolic and end-diastolic volumes were calculated according to the "method of discs" to derive LVEF (normal LV systolic function defined as LVEF ≥50%)[10]. LV mass was measured by the area-length method and indexed by height^2.7 ^(LVM/Ht^2.7^) to adjust for body habitus[11]. LVH was defined as LVM/Ht^2.7 ^>51 g/m^2.7 ^for men and >49.5 g/m^2.7 ^for women[12]. Pulse-wave Doppler (PWD)-derived transmitral indices were recorded from the four-chamber view at the mitral valve leaflet tips to determine the early diastolic transmitral (E-wave) and atrial (A-wave) velocities (m/s), the E/A wave velocity ratio, E-wave deceleration time (DT, in ms); the isovolumic relaxation time (IVRT, in ms) was measured by continuous-wave Doppler from the apical 5-chamber view[13]. Tissue Doppler Imaging (TDI)-derived early diastolic myocardial velocity was obtained from a 2.5 mm sample at the septal and lateral mitral annulus in the apical four-chamber view (E'_sep _and E'_lat_, A'_sep _and A'_lat_, respectively, in cm/s) with the average value represented as a "global" value (E'_gl _and A'_gl_, in cm/s) [13-15]. The mitral E-wave/E' ratio was calculated to estimate the LV filling pressure (E/E'_sep_, E/E'_lat_, and E/E'_gl_)[16,17]. All reported measurements represent the average of three consecutive cardiac cycles obtained by a single observer blinded to clinical status. The intra-class correlation coefficients for echocardiographic indices measured in our lab are: 0.75-0.88 for LV structure (i.e., LV mass/volumes) and 0.82-0.97 for PWD- and TDI-derived indices of LV diastolic function (i.e., E-, A-waves, DT, IVRT, and E').

### III. *PPAR*-complex genes genotyping and quality control

SNPs in three *PPAR*-complex genes (i.e., *PPARA*, *PPARG*, and *PPARGC1A*) were genotyped using Illumina BeadArray technology; 22 *PPARA*, 17 *PPARG*, and 39 *PPARGC1A *were selected to provide dense coverage and for compatibility in the multiplex reactions. Excluded from analysis were SNPs a) that deviated from Hardy-Weinberg equilibrium (HWE, p < 0.01), b) with minor allele frequency ≤5%, and/or c) with genotype call rates ≤90%. Maximal tolerated individual missing rate was 50%.

### IV. Statistical analysis

#### A. Extract latent LVDF trait by ICA

Initial quality control was performed on each of the echocardiography-derived endophenotypes by univariate analysis; those with an absolute skewness >1.5 or absolute kurtosis >2.0 were log-transformed for the factor analysis. Potential confounding effects were adjusted by regression of the values over a cubic age polynomial within sex group; residuals from the regression were used for ICA and further analyses.

We used a freely available implementation called *FastICA *(version 1.9) available in *R *(v. 2.7.0) to analyze the residuals of a matrix of the echocardiography-derived endophenotypes by pre-specified numbers of latent components[8,18,19]. For each component, the output from ICA consists of the extracted independent component (IC) represented by column vectors of the matrix of loadings on the echocardiography-derived endophenotypes and the coefficients of the extracted IC for each subject. The coefficients were then used to represent the individual latent LVDF trait values in subsequent analyses. Spearman's correlation between several traditional risk factors (systolic and diastolic blood pressure, body mass index, insulin, total cholesterol, triglycerides, LDL-C, HDL-C) and the latent LVDF trait were performed.

#### B. Select latent LVDF trait from extracted ICs

Loadings of each IC were examined for overrepresentation of the echocardiography-derived endophenotypes, which may reflect the importance of a particular IC. Individual IC's were treated as a potential latent LVDF trait and its clinical characteristics were characterized by examining risks of LVDF in cohorts defined by the median of corresponding IC coefficients (groups assigned as "High-Risk" and "Low-Risk"). The means of each of the 14 echocardiography-derived phenotypes were compared to gauge the IC's capability of separating risks. Three expert echocardiographers (JMJ, LdlF, VGD-R) independently assessed the distributional characteristics of these measures and selected the IC that yielded the most clinically relevant separation between groups; by consensus the IC was selected to represent the latent LVDF trait for subsequent analysis.

#### C. Genetic association analysis of the selected latent LVDF trait

HWE was tested by χ^2 ^test with 1 degree of freedom to identify SNPs with potential systematic bias resulting from genotyping errors. Linear regression analysis was performed to test for associations between the selected latent LVDF trait (represented by the IC coefficient) and the *PPAR*-complex candidate SNPs. In each regression model, covariates for age, sex, hypertension status, and LVM/Ht^2.7 ^were included. All regression analyses of genetic association were performed by SAS (v. 9.1.3, SAS Institute Inc., Cary, NC).

#### D. Explore potential SNP-SNP (within-gene and gene-gene) interactions

For identification of SNP-SNP interactions of potential importance to LVDD, a two-step procedure was applied: Step 1) detection of interactions within individual candidate genes (within-gene), and Step 2) identification of between-gene interactions (gene-gene). To deal with the large number of variables representing individual SNPs, their interactions, and their potential for collinearity, ridge regression analysis was performed (*R *package *"penalized" *version 0.9); ridge regression effectively eliminates many variables that are irrelevant to the trait of interest and effectively eliminates the collinearity problem[20,21]. In Step 1, for each candidate gene, ridge regression was applied to a model including all SNPs within that gene and their pair-wise interactions. SNPs that had main and/or interaction effects selected by the ridge regression were retained. In Step 2, all retained SNPs and their pairwise interactions (including those between genes) were entered into a general model. Ridge regression was again applied to the general model to identify a final set of important SNPs and interactions that together is significantly associated with the LVDD trait. Finally, conventional linear regression was applied to the selected SNPs and interactions to derive the significance levels of the associations.

#### E. Statistical power and adjustment for multiple testing

Quanto (v.1.2, http://hydra.usc.edu/gxe) was used to estimate statistical power (set at 80% at a significance level of 0.05 with a 2-sided alternate hypothesis)[22]. The power calculation assumed that an associated SNP has minor allele frequency of 0.2 and a locus-specific heritability of at least 2%. To adjust for multiple testing, the method of false discovery rate was applied in SAS.

## Results

### I. Clinical and echocardiographic characteristics

The clinical and echocardiographic characteristics are shown in Tables 1 and 2, respectively. The majority of subjects had normal LV systolic function; mild LV systolic dysfunction (LVEF 40-49%) was present in only 1.9% of the population; 17% of subjects had LVH. Distributional characteristics of the variables are shown in Additional File 1, Table S1.

**Table 1:** Clinical characteristics of study subjects (n = 403).

Age (yrs)	50 ± 13
BMI (kg/m^2^)	30 ± 6
Male, n (%)	187 (46)
SBP (mmHg)	123 ± 16
DBP (mmHg)	79 ± 9
Heart rate (beats per min)	66 ± 11
Diabetes mellitus, n (%)	57 (14)
Hypertension, n (%)	147 (37)
Creatinine (mg/dL)	0.9 ± 0.2
Glucose (mg/dL)	95 ± 27
Total Cholesterol (mg/dL)	195 ± 36
Triglycerides (mg/dL)	140 ± 93
LDL-C (mg/dL)	115 ± 31
HDL-C (mg/dL)	53 ± 15

Values represent the means ± 1 standard deviation or the number (%). BMI, body mass index; DBP, diastolic blood pressure; HDL-C, high density lipoprotein cholesterol; LDL-C, low density lipoprotein cholesterol; SBP, systolic blood pressure.

**Table 2:** Echocardiographic measurements of study population (n = 403).

LVEF (%)	64 ± 6
LVM/Ht^2.7 ^(g/m^2.7^)	41.5 ± 10.1
E-wave (m/s)	0.72 ± 0.16
A-wave (m/s)	0.57 ± 0.17
E/A ratio	1.35 ± 0.48
DT (ms)	207 ± 38
IVRT (ms)	94 ± 18
LA diameter (cm)	3.9 ± 0.5
E'_sep _(cm/s)	10.1 ± 2.7
E'_lat _(cm/s)	13.2 ± 3.5
E'_gl _(cm/s)	11.7 ± 2.9
A'_sep _(cm/s)	10.8 ± 1.7
A'_lat _(cm/s)	10.8 ± 2.2
A'_gl _(cm/s)	10.8 ± 1.7
E/E'_sep_	7.5 ± 2.4
E/E'_lat_	5.8 ± 2.0
E/E'_gl_	6.5 ± 2.1
LV diastolic septal wall (cm)	1.0 ± 0.2
LV diastolic posterior wall (cm)	1.0 ± 0.1
LV systolic diameter (cm)	3.2 ± 0.5
LV diastolic diameter (cm)	5.0 ± 0.5
% of subjects with LVH	17.4%*

A, late diastolic mitral flow velocity; DT, deceleration time; E, early diastolic mitral flow velocity; EF, ejection fraction; E'_gl_, E'_lat_, E'_sep_, early diastolic mitral annular velocity (global, lateral, and septal, respectively); E/E'_gl_, E/E'_lat_, E/E'_sep_, ratio of early mitral flow to the early mitral annular velocity (global, lateral, and septal, respectively); IVRT, isovolumic relaxation time; LA, left atrial; LV, left ventricular.

* male 18.1%, female 16.6%

### II. Extraction and identification of latent LVDF components

**Figure 1:**
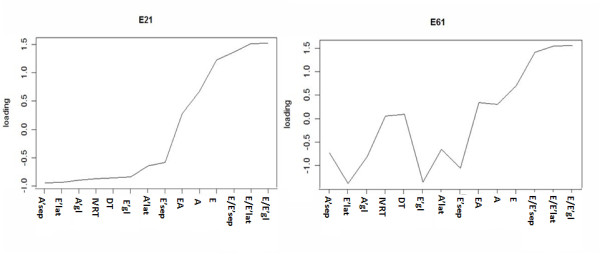
**Plots of echocardiography-derived phenotype loadings of two latent LVDD-related components, E21 (left) and E61 (right)**. In order to facilitate comparisons, the phenotypes on both graphs were reordered from lowest to highest according to the loadings of E21 (chosen arbitrarily). For comparison, note that E61 has higher loadings for both LV filling indices (i.e., E/E') and LV relaxation indices (i.e., E'); in contrast E21 exhibits similar loadings for LV relaxation indices but much lower loadings for LV relaxation, thus E61 is the selected component.

ICA analyses were performed by extracting 2, 3, and 6 components. Each extracted latent LVDF component was denoted by a letter "E", followed by a first digit which denoted the predefined number of components, and a secondary digit which denoted the individual component. For example, E21 refers to the first extracted component of a total of 2 latent components (Figure 1; figures for all extracted components in Additional File 1, Figures S1-S3). The collective loading pattern across the echocardiography-derived endophenotypes reflects the capability of a component to summarize a common source of regulation or an underlying interaction observed among genes. The component E61 had the highest loadings on E'_sep_, E'_lat_, E'_gl_, E/E'_sep_, E/E'_lat_, and E/E'_gl _compared to all other components in the loading of plots (Figure 1) and was thus selected as the latent LVDF endophenotype (Additional File 1, Table S2). The Spearman's correlation coefficients between several traditional risk factors (systolic and diastolic blood pressure, body mass index, insulin, total cholesterol, triglycerides, LDL-C, HDL-C) and the latent LVDF trait were determined (Additional File 1, Table S3). With the exception of LDL-C and total cholesterol, the remaining risk factors were significantly correlated with the latent LVDF trait.

### III. Ascertainment of quality of selected latent LVDF trait

The entire cohort was grouped as "High Risk" or "Low Risk" based on the medians of the individual latent LVDF trait values (i.e. individual ICs corresponding to each extracted component). Clinical characteristics and echocardiographic measurements in the "High-risk" and "Low-risk" groups are shown in Table 3 and Table 4, respectively. E61 resulted in the highest loadings on the TDI-derived echocardiographic variables, and thus, resulted in the most distinct separation of these LVDF echocardiography-derived measures (Additional File 1, Tables S4-6).

**Table 3:** Clinical characteristics of the high-risk (n = 201) and low-risk groups (n = 202) using E61.

	Low-Risk Group	High-Risk Group
Age (yrs)	49.4 ± 13.0	50.6 ± 12.0
BMI (kg/m^2^)	31.5 ± 5.5	28.2 ± 6.4
Male, n (%)	88 (44)	99 (49)
SBP (mmHg)	125 ± 17	121 ± 16
DBP (mmHg)	81 ± 9	77 ± 8
Heart rate (beats per min)	67 ± 11	65 ± 11
Diabetes mellitus, n (%)	39 (19)	18 (9)
Hypertension, n (%)	81 (40)	66 (33)
Creatinine (mg/dL)	0.8 ± 0.2	0.9 ± 0.2
Glucose (mg/dL)	98 ± 31	92 ± 23
Total Cholesterol (mg/dL)	197 ± 35	193 ± 36
Triglycerides (mg/dL)	150 ± 85	129 ± 99
LDL-C (mg/dL)	116 ± 31	112 ± 31
HDL-C (mg/dL)	51 ± 14	56 ± 15

Values represent the means ± 1 standard deviation or the number (%). BMI, body mass index; DBP, diastolic blood pressure; HDL-C, high density lipoprotein cholesterol; LDL-C, low density lipoprotein cholesterol; SBP, systolic blood pressure.

**Table 4:** Descriptive statistics for primary echocardiographic endophenotypes in E61.

	Low-Risk Group	High-Risk Group
E (m/s)	0.67 ± 0.14	0.76 ± 0.16
A (m/s)	0.52 ± 0.15	0.63 ± 0.18
E/A	1.41 ± 0.52	1.30 ± 0.42
DT (ms)	210 ± 39	204 ± 37
IVRT (ms)	95 ± 19	92 ± 18
E'_lat_(cm/s)	14.7 ± 3.3	11.8 ± 3.1
E'_sep_(cm/s)	11.1 ± 2.8	9.1 ± 2.1
E'_gl_(cm/s)	12.9 ± 2.9	10.4 ± 2.4
A'_lat_(cm/s)	10.6 ± 2.3	11.0 ± 2.0
A'_sep_(cm/s)	10.8 ± 1.8	10.7 ± 1.6
A'_gl_(cm/s)	10.7 ± 1.8	10.9 ± 1.5
E/E'_lat_	4.7 ± 1.0	6.9 ± 2.2
E/E'_sep_	6.3 ± 1.3	8.7 ± 2.5
E/E'_gl_	5.3 ± 1.1	7.6 ± 2.2

Values represent the means ± 1 standard deviation. A, late diastolic transmitral inflow velocity; A'_gl_, A'_lat_, A'_sep_, late diastolic mitral annular velocity (global, lateral, and septal, respectively); DT, deceleration time of transmitral E wave; E, early diastolic transmitral inflow velocity; E'_gl_, E'_lat_, E'_sep_, early diastolic mitral annular velocity (global, lateral, and septal, respectively); E/E'_gl_, E/E'_lat_, E/E'_sep_, ratio of early transmitral flow to the early mitral annular velocity (global, lateral, and septal, respectively); IVRT, isovolumic relaxation time.

### IV. Genetic association analysis of the latent LVDF trait

After applying exclusion criteria, the resultant dataset consisted of 64 SNPs (15 *PPARA*, 14 *PPARG*, and 34 *PPARGC1A*). Individual locus information including allele frequencies and HWE p-values for all 78 SNPs (Additional File 1, Tables S6A-C) and the Haploview linkage disequilibrium displays for each gene (Additional File 1, Figures S4-6) are shown in the supplement. By linear regression analysis of single SNPs (after adjustment for covariates), 7 SNPs (4 in *PPARA*, 2 in *PPARGC1A*, 1 in *PPARG*) were significantly associated with the latent LVDF trait (E61), whereas a range of 0 to 4 SNPs were associated with each of the 14 echocardiography-derived endophenotypes (Table 5). The frequency distribution of the P values derived from the latent LVDF trait exhibited a higher degree of statistical significance than those derived from the echocardiography-derived endophenotypes (Additional File 1, Figure S7).

**Table 5:** P-values of genetic association study for candidate SNPs with lent LVDD trait: ICA and 14 echocardiography-derived endophenotypes.

Locus	Gene context	ICA E61	Risk allele	Echocardiography-derived LVDF endophenotypes													
				
				E	A	E/A	DT	IVRT	E'_sep_	E'_lat_	E'_gl_	E/E'_sep_	E/E'_lat_	E/E'_gl_	A'_sep_	A'_lat_	A'_gl_
***PPARGC1A***																	

rs12500214	I-2				0.01					0.02	0.04				0.026	0.028	0.009
rs3736265	E-9						0.03										
rs3755862	I-7	0.038	A				0.03										
rs3774902	I-1	0.034	T														
rs3774921	I-10			0.021													
rs768695	I-12															0.011	0.018
rs7672915	I-2											0.049					
rs7677000	I-2									0.04							

***PPARA***																	

rs4253623	I-1A	0.045	G				0.04										
rs4253655	I-B															0.028	0.043
rs4253681	I-2A	0.039	C												0.005		0.009
rs4253725	I-3			0.042												0.044	
rs4253760	I-6	0.021	G				0.04						0.02				
rs4253765	I-6	0.02	C										0.02				

***PPARG***																	

rs1797912	I-1											0.026					
rs2972162	I-4				0.04								0.02	0.029			
rs3856806	E-7	0.006	T														

E-, exon; I-, intron. Only p values < 0.05 are shown.

### V. Within-gene and gene-gene interactions

**Figure 2:**
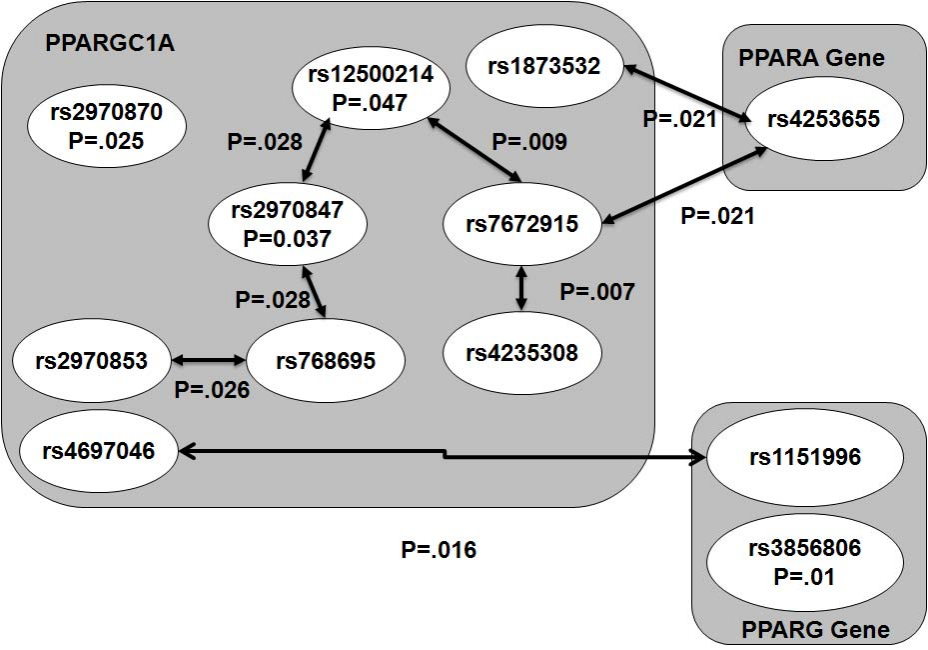
**Significant SNPs and their interactions (within-gene and gene-gene), with corresponding p-values identified by the two-step ridge regression analysis are shown**. SNPs are grouped by the candidate genes (i.e., *PPARGC1A*, *PPARA *and *PPARG*); significant interactions are shown by bidirectional arrows between two interacting SNPs: within-gene (arrows within boxes) and gene-gene interactions (arrows between boxes).

In the within-gene analysis, significant SNPs (8 in *PPARA*, 12 in *PPARGC1A*, and 4 in *PPARG*) for each of the three genes were retained for the final best-fit model (log likelihood: -276.1, -281.2 and -281.6, respectively; Table 6 and Figure 2). *PPARGC1A *SNPs rs12500214 significantly interacted with both rs2970847 and rs7672915 (p = 0.02 and 0.009, respectively); rs768695 significantly interacted with both rs2970847 and rs2970853 (both p = 0.03); and rs4235308 significantly interacted with rs7672915 (p = 0.007). No significant within-gene interactions were found among *PPARA *and *PPARG *SNPs.

**Table 6:** Significant Gene-Gene interactions in three *PPAR*-complex genes with latent LVDD endophenotype (E61).

Gene	Locus	Gene Context	Main Effect	P Value	Within-Gene Interaction	P value	Gene-Gene Interaction	P Value
*PPARGC1A*	rs12500214	Intron 2	Y	0.047	Y (rs2970847)	0.0278	N	0.0094
*PPARGC1A*	rs7672915	Intron 2	N		Y (rs12500214)	0.0092	N	
*PPARGC1A*	rs2970847	Exon 8	Y	0.037	Y (rs768695)	0.0276	N	
*PPARGC1A*	rs768695	Intron 12	N		Y (rs2970853)	0.0262	N	0.0318
*PPARGC1A*	rs4235308	Intron 2	N		Y (rs7672915)	0.0068	N	
*PPARGC1A*	rs2970870	Promoter	Y	0.025	N		N	
*PPARG*	rs3856806	Exon 7	Y	0.010	N		N	
*PPARGC1A*	rs1873532	Intron 10	N		N		Y (*PPARA*, rs4253655)	0.0214
*PPARGC1A*	rs4697046	Intron 2	N		N		Y (*PPARG*, rs1151996)	0.0163
*PPARGC1A*	rs7672915	Intron 2	N		N		Y (*PPARA*, rs4253655)	0.0212

N, no; Y, yes.

In the gene-gene analysis, rs4253655 in *PPARA *significantly interacted with rs1873532 (p = 0.02) and rs7672915 (p = 0.02), both in *PPARGC1A*. Significant interactions were also found between rs1151996 in *PPARG *and rs4697046 in *PPARGC1A *(p = 0.01). No gene-gene interactions were found between *PPARA *and *PPARG *genes.

### VI. Power calculation and false discovery rate

The study sample of 403 subjects provided adequate power to detect association of any SNP with a heritability ≥0.02 and an allele frequency ≥0.2 (power ≥81%, significance level α = 0.05). Although there was a significant association for several individual SNPs in single SNP analysis, none remained significant after adjustment for multiple testing by the False Discovery Rate.

## Discussion

In this study, we show genetic association between echocardiography-derived endophenotypes of LVDF and SNPs in three *PPAR*-complex genes involved in the transcriptional regulation of fatty acid metabolism. ICA was used to extract latent LVDF traits from 14 echocardiography-derived endophenotypes used clinically to evaluate diastolic function. We found that the latent LVDF trait identified relevant profiles of echocardiography-derived endophenotypes that could be clinically recognized as representing individuals of either High- or Low-Risk of LVDD. By use of linear regression analysis, 7 SNPs (4 in *PPARA*, 2 in *PPARGC1A*, 1 in *PPARG*) were significantly associated with the latent LVDF trait. In *PPARA*, 2 SNPs were clustered around intron 6 and two were in introns 1A and 2A, adjacent to alternate promoters. The significant *PPARGC1A *SNPs were located in introns 1 and 7. The single significant *PPARG *SNP (rs3856806) is a synonymous coding SNP in Exon 7 (H447H).

Complex phenotypes such as LVDF arise from multiple gene-gene and gene-environment interactions, each contributing a small effect to the overall expression of the trait. A consideration of epistasis, or gene-gene interactions, may identify a portion of the unexplained risk (i.e., "missing heritability" [23]) noted in the overwhelming majority of genetic studies of complex diseases. It is therefore of particular importance to investigate potential gene-gene interactions in the complex mechanisms responsible for LVDF. In the present study, an exploratory analysis of SNP-SNP interactions by ridge regression yielded potentially important findings. First, significant associations detected by modeling both main effects and interactions were largely different from those by main effects alone; with the only exception of rs3856806, which had a relatively strong marginal effect, suggesting that the single-SNP scan approach may only be appropriate for variants with strong signals. Second, all gene-gene interactions involved *PPARGC1A*, probably reflecting the important regulatory role of this gene. Thus, metabolic modulation of left ventricular diastolic function by *PPAR*-complex genes may be an important mechanism in the development of LVDD and may contribute to the pathogenesis of DHF. This is the first study to show a genetic association between LVDF and *PPAR*-complex genes in humans.

### Regulation of myocardial metabolism and cardiac structure/function by *PPAR-complex*

Alterations in myocardial fatty acid metabolism have been shown in both animal models and in humans to be an important determinant of the presence and development of hypertensive heart disease-related traits including hypertension, LVH, LVSD, and LVDD[3,24]. The normal fasting adult mammalian heart derives approximately two-thirds of ATP from the oxidation of fatty acids[25]. Under normal physiologic conditions, the heart is able to switch the energy substrate utilization from fatty acid to carbohydrates during the fed state, however under pathologic conditions the heart loses this metabolic plasticity[26]. For example, our group has previously shown that fatty acid metabolism is decreased in patients with heart failure and is a strong predictor of LV mass in patients with hypertension and in normotensive controls[27]. Metabolic modulation of the heart occurs primarily at the transcriptional level through the coordinated regulation of enzymes and proteins in specific metabolic pathways. *PPAR*-complex genes, transcription factors and coactivators known to regulate the expression of fatty acid transport and oxidation genes, have been shown in elegant transgenic models to modulate the pathophysiology of LV systolic and diastolic dysfunction[3,28-36]. The *PPARA *gene has been identified as a "master switch" for the metabolic remodeling of the heart, particularly when coactivated by *PPARGC1A *[36-39]. *PPARA *expression is down-regulated in the adapted, hypertrophied pressure-overloaded heart and reactivation of *PPARA *by an agonist is associated with contractile dysfunction[40]. Cardiac-restricted overexpression of *PPARA *results in hypertrophy, the activation of gene markers of pathologic hypertrophic growth, and in systolic dysfunction[32]. *PPARG*, on the other hand, plays a critical role in modulating the substrate environment of the heart by its actions in adipocytes[41]. Myocardial fatty acid metabolism has also been more specifically associated with indices of LV diastolic function in animal models and in humans[1-3,42,43]. In an animal study, *PPARA *reduced myocardial fibrosis and prevented the development of diastolic dysfunction[44]. Ciglitazole (a *PPARG *agonist) has also been shown to attenuate LVDD in pressure-overloaded rats[45]. *PPARGC1A *is a key regulator of cardiac energy metabolism; overexpression of *PPARGC1A *in the murine heart leads to a modest increase in mitochondrial number, derangements of mitochondrial ultrastructure, and development of cardiomyopathy[32,46]. Thus, there is ample evidence to support a critical role for *PPAR*-complex genes in regulating cardiac structure and function. However, the precise molecular mechanisms by which these alterations may modulate LVDF remain uncertain.

Several lines of evidence support the existence of genetic association between LVDF and *PPAR*-complex genes in humans. Recent reports have shown associations between cardiovascular traits and 4 of the 19 SNPs indentified in the present study. The most widely published among these, a synonymous coding *PPARGC1A *SNP rs2970847 (T394T), has been associated individually, in combination with other *PPARGC1A *SNPs in haplotype blocks, and in gene-gene interactions with relevant traits including diabetes risk, glucose uptake, obesity, and DNA damage in a variety of populations [47-54]. Intriguingly, this SNP has also been associated with non-hypertensive LVH in a cohort including 270 hypertrophic cardiomyopathy and 2486 hypertensive patients (with and without LVH), yielding an odds ratio 1.49 (95% confidence interval, 1.15-1.98)[55]. The significant *PPARGC1A *SNP rs4697046 has also been linked with plasma glucose and DNA damage[56]. In our study, this SNP was found to interact with two other *PPARGC1A *SNPs, although these SNPs were not found to share significant linkage disequilibrium (data not shown), thus implicating *cis*-acting regulatory elements.

This study also identified two SNPs (rs3856806 in *PPARG *and rs4253623 in *PPARA*) as associated with MI risk [57-59]. The rare allele of *PPARG *SNP rs3856806 has been linked with not only coronary artery disease progression, but also with pro-inflammatory cytokines (MMP9 and TNFα), plasma homocysteine levels, and obesity [60-62]. The minor allele of *PPARA *alternate promoter SNP, rs4253623, along with another significant *PPARA *promoter SNP rs4253681, is clustered adjacent to alternatively-spliced untranslated exons 1A and 2A and may play a role in regulating *PPARA *gene expression by mediating the expression of the 6 alternative spliced variants resulting from 4 different promoters[63]. Thus, it is plausible that variants in *PPAR*-complex genes may modulate LVDF-related traits in humans. However, all these possible mechanisms deserve to be explored further in animal models.

### Utility of ICA for study of genetic association

The present study was performed using state-of-the-art analysis techniques in genetic epidemiology and represents a continuation of our study of the genetics of hypertensive heart disease through analysis of endophenotypes, which lay proximal along the pathway from observed clinical phenotype to genotype[8]. The latent factor analysis by ICA was used to address the multidimensional data in which non-Gaussian structure such as clustering and independence represent important components. Although several echocardiographic measures are considered jointly to clinically ascertain LVDF, the precise diagnosis of LVDD remains controversial and there currently exists no universally accepted diagnostic criteria[4,64,65]. Furthermore, many echocardiographic endophenotypes are characterized by age-dependency and non-linear associations with disease severity, presenting additional challenges for genetic association studies of this common, complex disease. The ICA trait is extracted from a panel of measured echocardiography-derived endophenotypes, which themselves represent traits more proximal to the genetic underpinnings, and as such has emerged as an important tool in the study of complex diseases[66,67]. ICA is well-suited to the study of LVDF because it effectively reduces the number of dimensions by identifying linear representations of the original endophenotypes. The ICA-derived latent LVDF trait showed significant associations with 7 *PPAR*-complex SNPs, whereas a range of 0 to 4 SNPs were associated with each of the 14 echocardiography-derived endophenotypes. The frequency distribution of P values showed a greater proportion of significant associations with the latent LVDF trait than for the measured echocardiography-derived endophenotypes, suggesting that analyses of the latent trait improved detection of the genetic underpinnings of LVDF. Thus, use of ICA-derived latent LVDF trait improved our ability to detect the genetic underpinning of LVDD, as our group and others have previously shown for other echocardiography-derived endophenotypes[8,68].

The ridge regression method was used to handle collinearity among SNPs, and thus allowed for testing many SNPs simultaneously. Since these three *PPAR*-complex genes are all related to fatty acid metabolism, and LVDD is a complex disease, epistasis may contribute to LVDF. Thus, there was a real possibility of collinearity among SNPs, a result of multiple makers exhibiting strong linkage disequilibrium (LD) in a single genomic region. The presence of LD between SNPs at neighboring loci can make it difficult to distinguish functionally relevant variations from nonfunctional variations. However, the application of ridge regression to the evaluation of SNP-SNP interactions provided a robust method to control for the effect of collinearity.

Finally, we investigated within-gene and gene-gene SNP-SNP interactions using a two-stage procedure as outlined in the methods. This led to interesting findings of interaction effects that were not detected by testing for marginal effects alone. This last issue is of particular importance as supported by recent studies in yeast showing that "pure" interactions (with little or negligible marginal effects) are both real and important[69].

## Conclusions

ICA-derived latent LVDF traits improved the ability to detect genetic underpinnings of LVDF. Significant genetic associations were found between *PPAR*-complex gene variants and LVDF, suggesting that these genes may be involved in the pathogenesis of this complex disease. Although these findings of association are suggestive, these results need to be validated by mechanistic studies in animal models. Pharmacologic modulation of myocardial substrate environment, *PPAR*-complex genes, and/or both may be an important target to restore myocardial metabolic function and thus normalization of left ventricular diastolic function.

## Abbreviations

(DHF): diastolic heart failure; (DT): deceleration time; (HHD): hypertensive heart disease; (HF): heart failure; (HWE): Hardy-Weinberg equilibrium; (IC): independent components; (ICA): independent component analysis; (IVRT): isovolumic relaxation time; (LD): linkage disequilibrium; (LV): left ventricular; (LVDF): left ventricular diastolic function; (LVH): Left Ventricular hypertrophy; (LVSD): left ventricular systolic dysfunction; (*PPAR*): peroxisome proliferator-activated receptor; (PWD): Pulse-wave Doppler; (SNPs): single nucleotide polymorphisms; (TDI): Tissue Doppler Imaging.

## Competing interests

The authors declare that they have no competing interests.

## Authors' contributions

JMJ conceived and designed the study, performed statistical analyses, interpret the data, and drafted the manuscript. LdlF conceived and designed the study, performed statistical analyses, warranted the quality of the genetic data, interpret the data, and provided a substantive review of the manuscript. ADW conceived and designed the study, collected and warranted the quality of the echocardiographic phenotype data and provided a substantive review of the manuscript. CG performed statistical analyses, interpret the data, and provided a substantive review of the manuscript. VGD-R conceived and designed the study, interpret the data, offered the guidance on the whole study and provided a substantive review of the manuscript. All authors have read and approved the final manuscript.

## Pre-publication history

The pre-publication history for this paper can be accessed here:

http://www.biomedcentral.com/1471-2350/11/65/prepub

## Supplementary Material

Additional file 1 **Supplementary Tables and Figures**. Table S1. Eleven LVDD-related echocardiographic parameters in the ICA analysis. Table S2. Absolute echocardiographic parameter loadings of 2-, 3-, 6-component ICA. Table S3. Spearman's correlation coefficients between traditional cardiovascular risk factors and the latent LVDF trait. Table S4. Descriptive statistics for primary echocardiographic endophenotypes (2 components set). Table S5. Descriptive statistics for primary echocardiographic endophenotypes (3 components set). Table S6. Descriptive statistics for primary echocardiographic endophenotypes (6 components set). Table S7A-C. Characteristics of 39 SNPs in the *PPARGC1A*, *PPARA*, and *PPARG *genes. Figure S1. Plots of echocardiographic endophenotype loadings of E21 and E22. Figure S2. Plots of echocardiographic endophenotype loadings of E31, E32 and E33. Figure S3. Plots of echocardiographic endophenotype loadings of E61-E66. Figure S4. Haploview LD display of 34 *PPARGC1A *SNPs. Figure S5. Haploview LD display of 15 *PPARA *SNPs. Figure S6. Haploview LD display of the 14 *PPARG *SNPs. Figure S7. Comparison of the distributions of P values between latent LVDD trait (E61) and primary 14 echocardiographic endophenotypes. Click here for file
