# Supplementary material for: Association and interaction of PPAR-complex gene variants with latent traits of left ventricular diastolic function
Source: BMC Med Genet. 2010 Apr 28;11:65. doi: 10.1186/1471-2350-11-65 (PMC2874543; doi:10.1186/1471-2350-11-65)
Supplement: Additional file 1 — Supplementary Tables and Figures. Table S1. Eleven LVDD-related echocardiographic parameters in the ICA analysis. Table S2. Absolute echocardiographic parameter loadings of 2-, 3-, 6-component ICA. Table S3. Spearman's correlation coefficients between traditional cardiovascular risk factors and the latent LVDF trait. Table S4. Descriptive statistics for primary echocardiographic endophenotypes (2 components set). Table S5. Descriptive statistics for primary echocardiographic endophenotypes (3 components set). Table S6. Descriptive statistics for primary echocardiographic endophenotypes (6 components set). Table S7A-C. Characteristics of 39 SNPs in the PPARGC1A, PPARA, and PPARG genes. Figure S1. Plots of echocardiographic endophenotype loadings of E21 and E22. Figure S2. Plots of echocardiographic endophenotype loadings of E31, E32 and E33. Figure S3. Plots of echocardiographic endophenotype loadings of E61-E66. Figure S4. Haploview LD display of 34 PPARGC1A SNPs. Figure S5. Haploview LD display of 15 PPARA SNPs. Figure S6. Haploview LD display of the 14 PPARG SNPs. Figure S7. Comparison of the distributions of P values between latent LVDD trait (E61) and primary 14 echocardiographic endophenotypes. [file 1471-2350-11-65-S1.DOC]

**ONLINE SUPPLEMENT**

**Association and Interaction of PPAR-complex Gene Variants with Latent Traits of Left Ventricular Diastolic Function**

#### Jyh Ming Juang*, MD, MS^1,2^

#### Lisa de las Fuentes*, MD, MS^1^

#### Alan D. Waggoner, MHS^1^

#### C. Charles Gu, PhD^2,3^

#### Víctor G. Dávila-Román, MD, FACC, FASE^1^

From the ^1^Cardiovascular Imaging and Clinical Research Core Laboratory, Cardiovascular Division, ^2^Division of Biostatistics, and ^3^Department of Genetics, Washington University School of Medicine, St. Louis, Missouri.

* Drs. Jyh Ming Juang and Lisa de las Fuentes contributed equally to this work.

Short title: PPAR-complex Gene Variants and Latent LVDF Trait

**Address for correspondence:**

Víctor G. Dávila-Román, MD, FACC, FASE

Director, Cardiovascular Imaging and Clinical Research Core Laboratory

Cardiovascular Division, Box 8086

Washington University School of Medicine,

660 South Euclid Avenue, St. Louis, MO 63110

Tel: 314-362-4748

Fax: 314-747-8170

Email: [vdavila@wustl.edu](mailto:vdavila@wustl.edu)

**Supplementary Tables and Figures**

| **Table S1. Eleven LVDD-related echocardiographic parameters in the ICA analysis (n=403).** | | | |
| --- | --- | --- | --- |
|  | Mean ± SD | Skewness | Kurtosis |
| E (m/s) | 0.72 ± 0.16 | 0.3 | -0.1 |
| A (m/s) | 0.57 ± 0.17 | 0.7 | 0.2 |
| E/A | 1.35 ± 0.48 | 1.0 | 1.1 |
| DT (ms) | 211 ± 41 | 1.2 | 1.8 |
| IVRT (ms) | 97 ± 19 | 1.2 | **3.1** |
| E’_lat_ (cm/s) | 13.2 ± 3.5 | 0.5 | 0.6 |
| E’_sep_ (cm/s) | 10.1 ± 2.6 | 0.8 | 1.2 |
| E’_gl_ (cm/s) | 11.7 ± 2.9 | 0.7 | 1.0 |
| E/E’_lat_ | 5.8 ± 2.0 | **1.6** | **4.1** |
| E/E’_sep_ | 7.5 ± 2.4 | 1.5 | **3.6** |
| E/E’_gl_ | 6.5 ± 2.1 | 1.5 | **3.2** |
| A’_lat_ (cm/s) | 10.8 ± 2.2 | 0.28 | 0.07 |
| A’_sep_ (cm/s) | 10.8 ± 1.7 | 0.18 | -0.09 |
| A’_gl_ (cm/s) | 10.8 ± 1.7 | 0.13 | 0.06 |
| Skewness exceeding 1.5 and kurtosis exceeding 2.0 are highlighted in **bold** font. A, late diastolic transmitral inflow velocity; A’_gl_, A’_lat_, A’_sep_, late diastolic mitral annular velocity (global, lateral, and septal, respectively); DT, deceleration time of transmitral E wave; E, early diastolic transmitral inflow velocity; E’_gl_, E’_lat_, E’_sep_, early diastolic mitral annular velocity (global, lateral, and septal, respectively); E/E’_gl_, E/E’_lat_, E/E’_sep_, ratio of early transmitral flow to the early mitral annular velocity (global, lateral, and septal, respectively); IVRT, isovolumic relaxation time; SD, standard deviation. | | | |

| **Table S2. Absolute echocardiographic parameter loadings of 2-, 3-, 6-component ICA.** | | | | | | | | | | | | | | |
| --- | --- | --- | --- | --- | --- | --- | --- | --- | --- | --- | --- | --- | --- | --- |
|  | **E** | **A** | **E/A** | **DT** | **IVRT** | **E’_lat_** | **A’_lat_** | **E/E’_lat_** | **E’_sep_** | **A’_sep_** | **E/E’_sep_** | **E’_gl_** | **A’_gl_** | **E/E’_gl_** |
| **E21** | *1.22* | 0.66 | 0.27 | 0.85 | 0.87 | 0.94 | 0.64 | *1.51* | 0.59 | 0.94 | *1.36* | 0.84 | 0.89 | *1.52* |
| **E22** | 0.77 | 0.92 | *1.55* | 0.82 | 0.80 | *1.18* | 1.05 | 0.19 | *1.47* | 0.79 | 0.43 | *1.43* | *1.09* | 0.31 |
| **E31** | 0.04 | 0.87 | 0.40 | *1.29* | *1.37* | *1.01* | 0.92 | *1.01* | 0.89 | *1.09* | 0.99 | *1.07* | *1.21* | *1.03* |
| **E32** | *1.41* | 0.30 | *1.35* | *1.33* | *1.34* | 0.30 | *1.11* | 0.81 | 0.75 | *1.11* | 0.54 | 0.56 | *1.28* | 0.73 |
| **E33** | 0.56 | 0.70 | 0.69 | *1.35* | *1.45* | *1.08* | *1.07* | 0.82 | *1.07* | 0.70 | 0.89 | *1.14* | *1.12* | 0.91 |
| **E61** | **0.70** | **0.30** | **0.34** | **0.07** | **0.07** | ***1.38*** | **0.65** | ***1.54*** | ***1.06*** | **0.74** | ***1.41*** | ***1.36*** | **0.81** | ***1.55*** |
| **E62** | 0.74 | 0.10 | 0.10 | *3.56* | 0.19 | 0.16 | 0.26 | 0.32 | 0.43 | 0.14 | 0.21 | 0.32 | *0.28* | 0.31 |
| **E63** | 0.54 | 0.27 | 0.28 | 0.09 | 0.13 | *1.44* | 0.54 | 0.53 | 0.01 | *2.90* | 0.56 | 0.86 | *1.18* | 0.10 |
| **E64** | 0.96 | 0.24 | 0.25 | 0.17 | *3.53* | 0.19 | 0.20 | 0.21 | 0.27 | 0.16 | 0.13 | 0.28 | 0.28 | 0.20 |
| **E65** | 0.02 | *2.75* | *2.54* | 0.01 | 0.01 | 0.06 | 0.00 | 0.01 | 0.01 | 0.04 | 0.05 | 0.03 | 0.01 | 0.01 |
| **E66** | 0.39 | 0.36 | 0.40 | 0.10 | 0.12 | 0.08 | *2.60* | 0.51 | *1.67* | 0.35 | 0.69 | 0.85 | *1.54* | 0.01 |
| Significant deviations from 0 are noted in italics. **Bold** selected as the latent LVDF trait. | | | | | | | | | | | | | | |

| **Table S3. Spearman’s correlation coefficients between traditional cardiovascular risk factors and the latent LVDF trait.** | | |
| --- | --- | --- |
| **Latent LVDF trait** | **Correlation Coefficient** | ***P* value** |
| Systolic blood pressure | 0.178 | 0.0003 |
| Diastolic blood pressure | 0.205 | <0.0001 |
| Body mass index | 0.405 | <0.0001 |
| Insulin | 0.148 | 0.006 |
| Total cholesterol | -0.025 | 0.61 |
| Triglyceride | 0.169 | 0.0007 |
| LDL-C | 0.008 | 0.88 |
| HDL-C | -0.215 | <0.0001 |

| **Table S4. Descriptive statistics for primary echocardiographic endophenotypes (2 components set, each component and high-risk and low-risk groups).** | | | | | | | | | | | | | | |
| --- | --- | --- | --- | --- | --- | --- | --- | --- | --- | --- | --- | --- | --- | --- |
|  | **E** | **A** | **E/A** | **DT** | **IVRT** | **E’_lat_** | **E’_sep_** | **E’_gl_** | **A’_lat_** | **A’_sep_** | **A’_gl_** | **E/E’_lat_** | **E/E’_sep_** | **E/E’_gl_** |
|  | **(m/s)** | **(m/s)** |  | **(ms)** | **(ms)** | **(cm/s)** | **(cm/s)** | **(cm/s)** | **(cm/s)** | **(cm/s)** | **(cm/s)** |  |  |  |
| E21  Low Risk | 0.64±0.13 | 0.52±0.15 | 1.33±0.48 | 216±41 | 97±19 | 14.3±3.5 | 10.8±2.8 | 12.6±3.0 | 10.9±2.3 | 11.0±1.9 | 11.0±1.8 | 4.6±1.0 | 6.1±1.3 | 5.2±1.1 |
| E21  High Risk | 0.80±0.15 | 0.63±0.18 | 1.37±0.47 | 198±33 | 90±17 | 12.1±3.2 | 9.4±2.3 | 10.8±2.6 | 10.7±2.1 | 10.5±1.5 | 10.6±1.6 | 7.0±2.1 | 8.9±2.4 | 7.7±2.1 |
| **E22**  **Low Risk** | **0.76±0.15** | **0.52±0.16** | **1.57±0.48** | **197±31** | **89±16** | **14.5±3.4** | **11.3±2.7** | **12.9±2.9** | **10.1±2.0** | **10.5±1.7** | **10.3±1.6** | **5.6±1.9** | **7.1±2.2** | **6.2±1.9** |
| **E22**  **High Risk** | **0.67±0.16** | **0.62±0.17** | **1.14±0.36** | **216±42** | **98±20** | **12.0±3.1** | **8.9±2.0** | **10.4±2.4** | **11.5±2.1** | **11.1±1.7** | **11.3±1.7** | **6.0±2.1** | **7.9±2.5** | **6.8±2.2** |
| All measurements shown are mean ± standard deviation. The rows shown in **bold** represent the most clinically relevant separation between groups among the 2 component models. | | | | | | | | | | | | | | |

| **Table S5. Descriptive statistics for primary echocardiographic endophenotypes (3 components set, each component and high-risk and low-risk groups).** | | | | | | | | | | | | | | |
| --- | --- | --- | --- | --- | --- | --- | --- | --- | --- | --- | --- | --- | --- | --- |
|  | **E** | **A** | **E/A** | **DT** | **IVRT** | **E’_lat_** | **E’_sep_** | **E’_gl_** | **A’_lat_** | **A’_sep_** | **A’_gl_** | **E/E’_lat_** | **E/E’_sep_** | **E/E’_gl_** |
|  | **(m/s)** | **(m/s)** |  | **(ms)** | **(ms)** | **(cm/s)** | **(cm/s)** | **(cm/s)** | **(cm/s)** | **(cm/s)** | **(cm/s)** |  |  |  |
| E31  Low Risk | 0.69±0.14 | 0.53±0.16 | 1.41±0.50 | 205±36 | 93±17 | 14.4±3.4 | 11.0±2.7 | 12.8±2.9 | 11.2±2.3 | 11.2±1.8 | 11.2±1.8 | 5.0±1.3 | 6.5±1.5 | 5.6±1.3 |
| E31  High Risk | 0.74±0.17 | 0.61±0.18 | 1.30±0.44 | 208±40 | 94±20 | 12.0±3.2 | 9.1±2.2 | 10.6±2.6 | 10.4±1.9 | 10.4±1.5 | 10.4±1.5 | 6.6±2.3 | 8.5±2.6 | 7.4±2.3 |
| **E32**  **Low Risk** | **0.67±0.15** | **0.50±0.14** | **1.44±0.53** | **213±42** | **97±20** | **14.5±3.4** | **11.0±2.8** | **12.8±3.0** | **10.2±2.2** | **10.6±1.9** | **10.4±1.8** | **4.7±1.1** | **6.3±1.4** | **5.4±1.1** |
| **E32**  **High Risk** | **0.77±0.16** | **0.64±0.17** | **1.26±0.40** | **201±34** | **90±15** | **11.9±3.1** | **9.2±2.1** | **10.5±2.4** | **11.4±2.0** | **11.0±1.5** | **11.2±1.5** | **6.9±2.2** | **8.7±2.5** | **7.6±2.2** |
| E33  Low Risk | 0.81±0.14 | 0.57±0.18 | 1.55±0.50 | 193±30 | 87±15 | 13.7±3.7 | 10.8±2.8 | 12.3±3.1 | 10.1±2.0 | 10.3±1.6 | 10.2±1.5 | 6.3±2.2 | 8.0±2.6 | 7.0±2.2 |
| E33  High Risk | 0.63±0.13 | 0.57±0.16 | 1.16±0.35 | 220±41 | 100±19 | 12.7±3.3 | 9.4±2.3 | 11.1±2.6 | 11.5±2.1 | 11.3±1.7 | 11.4±1.6 | 5.3±1.7 | 7.0±2.1 | 6.0±1.8 |
| All measurements shown are mean ±standard deviation. The rows shown in **bold** represent the most clinically relevant separation between groups among the 3 component models. | | | | | | | | | | | | | | |

| **Table S6A. Descriptive statistics for primary echocardiographic endophenotypes (6 components set, each component and high-risk and low-risk groups).** | | | | | | | | | | | | | | |
| --- | --- | --- | --- | --- | --- | --- | --- | --- | --- | --- | --- | --- | --- | --- |
|  | **E** | **A** | **E/A** | **DT** | **IVRT** | **E’_lat_** | **E’_sep_** | **E’_gl_** | **A’_lat_** | **A’_sep_** | **A’_gl_** | **E/E’_lat_** | **E/E’_sep_** | **E/E’_gl_** |
|  | **(m/s)** | **(m/s)** |  | **(ms)** | **(ms)** | **(cm/s)** | **(cm/s)** | **(cm/s)** | **(cm/s)** | **(cm/s)** | **(cm/s)** |  |  |  |
| **E61**  **Low Risk** | **0.67±0.14** | **0.52±0.15** | **1.41±0.52** | **210±39** | **95±19** | **14.7±3.3** | **11.1±2.8** | **12.9±2.9** | **10.6±2.3** | **10.8±1.8** | **10.7±1.8** | **4.7±1.0** | **6.3±1.3** | **5.3±1.1** |
| **E61**  **High Risk** | **0.76±0.16** | **0.63±0.18** | **1.30±0.42** | **204±37** | **92±18** | **11.8±3.1** | **9.1±2.1** | **10.4±2.4** | **11.0±2.0** | **10.7±1.6** | **10.9±1.5** | **6.9±2.2** | **8.7±2.5** | **7.6±2.2** |
| E62  Low Risk | 0.78±0.15 | 0.58±0.18 | 1.47±0.52 | 199±34 | 82±11 | 13.5±3.5 | 10.6±2.7 | 12.1±3.0 | 10.9±2.3 | 10.8±1.7 | 10.9±1.7 | 6.2±2.0 | 7.8±2.3 | 6.8±2.1 |
| E62  High Risk | 0.65±0.14 | 0.56±0.16 | 1.23±0.40 | 214±41 | 104±18 | 12.9±3.5 | 9.5±2.5 | 11.2±2.9 | 10.7±2.1 | 10.8±1.7 | 10.7±1.6 | 5.4±2.0 | 7.2±2.4 | 6.1±2.1 |
| E63  Low Risk | 0.67±0.16 | 0.56±0.18 | 1.30±0.47 | 229±37 | 97±20 | 13.3±3.5 | 9.9±2.6 | 11.6±2.9 | 10.7±2.2 | 10.8±1.8 | 10.8±1.8 | 5.3±2.0 | 7.1±2.3 | 6.0±2.0 |
| E63  High Risk | 0.77±0.14 | 0.58±0.16 | 1.41±0.47 | 184±23 | 90±15 | 13.1±3.5 | 10.3±2.7 | 11.7±3.0 | 10.8±2.1 | 10.8±1.6 | 10.8±1.6 | 6.2±2.0 | 7.9±2.4 | 6.9±2.0 |
| All measurements shown are mean ±standard deviation. The rows shown in **bold** represent the most clinically relevant separation between groups among the 6 component models. | | | | | | | | | | | | | | |

| **Table S6B. Descriptive statistics for primary echocardiographic endophenotypes (6 components set, each component and high-risk/low-risk groups).** | | | | | | | | | | | | | | |
| --- | --- | --- | --- | --- | --- | --- | --- | --- | --- | --- | --- | --- | --- | --- |
|  | **E** | **A** | **E/A** | **DT** | **IVRT** | **E’_lat_** | **E’_sep_** | **E’_gl_** | **A’_lat_** | **A’_sep_** | **A’_gl_** | **E/E’_lat_** | **E/E’_sep_** | **E/E’_gl_** |
|  | **(m/s)** | **(m/s)** |  | **(ms)** | **(ms)** | **(cm/s)** | **(cm/s)** | **(cm/s)** | **(cm/s)** | **(cm/s)** | **(cm/s)** |  |  |  |
| E64  Low Risk | 0.69±0.16 | 0.58±0.17 | 1.27±0.46 | 211±38 | 94±18 | 13.2±3.6 | 9.9±2.5 | 11.6±2.9 | 11.6±2.1 | 12±1.3 | 11.9±1.4 | 5.6±1.8 | 7.2±2 | 6.2±1.8 |
| E64  High Risk | 0.74±0.16 | 0.56±0.18 | 1.44±0.48 | 203±38 | 93±18 | 13.2±3.5 | 10.3±2.8 | 11.8±3.0 | 9.9±1.9 | 9.6±1.1 | 9.8±1.3 | 6.0±2.2 | 7.7±2.6 | 6.7±2.3 |
| E65  Low Risk | 0.70±0.16 | 0.48±0.11 | 1.55±0.53 | 205±40 | 95±20 | 14.0±3.6 | 10.8±2.8 | 12.4±3.1 | 10.4±2.1 | 10.7±1.8 | 10.6±1.7 | 5.3±1.5 | 6.8±2.0 | 5.9±1.6 |
| E65  High Risk | 0.73±0.16 | 0.66±0.18 | 1.16±0.31 | 209±36 | 92±17 | 12.4±3.2 | 9.4±2.3 | 10.9±2.6 | 11.2±2.2 | 10.9±1.6 | 11.0±1.7 | 6.3±2.3 | 8.2±2.5 | 7.1±2.3 |
| E66  Low Risk | 0.69±0.15 | 0.58±0.16 | 1.26±0.42 | 209±36 | 94±16 | 13.1±3.3 | 9.7±2.3 | 11.4±2.7 | 12.3±1.7 | 11.4±1.6 | 11.9±1.3 | 5.6±1.8 | 7.5±2.2 | 6.3±1.9 |
| E66  High Risk | 0.74±0.16 | 0.56±0.19 | 1.44±0.51 | 205±40 | 93±20 | 13.3±3.7 | 10.5±2.9 | 11.9±3.2 | 9.2±1.4 | 10.2±1.6 | 9.7±1.3 | 6.0±2.2 | 7.5±2.6 | 6.6±2.3 |
| All measurements shown are mean ±standard deviation. The rows shown in **bold** represent clinically the most relevant separation between groups defined by the winning component (E61, component-1 in the 6-component analysis) that will be selected as the latent LVDD trait. | | | | | | | | | | | | | | |

| **Table S7A. Characteristics of 39 SNPs in the PPARGC1A gene.** | | | | | | |
| --- | --- | --- | --- | --- | --- | --- |
| Gene | Marker (refSNP) | Gene Context | Chromosomal Position | MAF (%) | HWE P-value | Call rate (%) |
| PPARGC1A | rs10938964 | INTRON 2 | 23449186 | 32.4 | 0.474 | 99.50 |
| PPARGC1A | rs12500214 | INTRON 2 | 23494666 | 24.4 | 0.062 | 99.75 |
| PPARGC1A | rs1472095 | INTRON 7 | 23427175 | 24.8 | 0.934 | 99.50 |
| PPARGC1A | rs16874284 | INTRON 2 | 23494593 | 0.7 | <0.001 | 99.75 |
| PPARGC1A | rs17574213 | EXON 8 | 23424779 | 7.8 | 0.714 | 99.75 |
| PPARGC1A | rs17576121 | PROMOTER | 23502372 | 27.7 | 0.054 | 99.50 |
| PPARGC1A | rs1873532 | INTRON 10 | 23415876 | 41.4 | 0.805 | 100 |
| PPARGC1A | rs2290603 | INTRON 5 | 23435531 | 22.1 | 0.877 | 99.50 |
| PPARGC1A | rs2932965 | INTRON 10 | 23414584 | 22.6 | 0.864 | 99.75 |
| PPARGC1A | rs2932967 | INTRON 8 | 23424223 | 22.1 | 0.839 | 99.75 |
| PPARGC1A | rs2932976 | INTRON 7 | 23431426 | 23.8 | 0.431 | 100 |
| PPARGC1A | rs2946385 | INTRON 2 | 23495421 | 44.6 | 0.072 | 99.50 |
| PPARGC1A | rs2946386 | PROMOTER | 23502677 | 22.8 | 0.957 | 96.28 |
| PPARGC1A | rs2946390 | PROMOTER | 23510057 | 34.8 | 0.545 | 99.75 |
| PPARGC1A | rs2970847 | EXON 8 | 23425022 | 20.1 | 0.794 | 99.50 |
| PPARGC1A | rs2970848 | INTRON 7 | 23426125 | 30.5 | 0.892 | 99.50 |
| PPARGC1A | rs2970849 | INTRON 7 | 23426905 | 32.2 | 0.909 | 99.50 |
| PPARGC1A | rs2970853 | INTRON 7 | 23432617 | 25.9 | 0.980 | 99.75 |
| PPARGC1A | rs2970869 | PROMOTER | 23502467 | 23.0 | 0.520 | 99.75 |
| PPARGC1A | rs2970870 | PROMOTER | 23502115 | 42.8 | 0.080 | 99.75 |
| PPARGC1A | rs3736265 | EXON 9 | 23423805 | 5.3 | 0.883 | 99.75 |
| HWE, Hardy-Weinberg equilibrium; MAF, minor allele frequency. | | | | | | |

| **Talble S7A. (continued). Characteristics of 39 SNPs in the PPARGC1A gene.** | | | | | | |
| --- | --- | --- | --- | --- | --- | --- |
| Gene | Marker (refSNP) | Gene Context | Chromosomal Position | MAF (%) | HWE P-value | Call rate (%) |
| PPARGC1A | rs3755862 | INTRON 7 | 23425935 | 5.2 | 0.922 | 99.75 |
| PPARGC1A | rs3755863 | EXON 8 | 23424620 | 40.8 | 0.984 | 99.75 |
| PPARGC1A | rs3774902 | INTRON 1 | 23499880 | 4.9 | 0.953 | 99.75 |
| PPARGC1A | rs3774921 | INTRON 10 | 23419745 | 47.0 | 0.093 | 99.50 |
| PPARGC1A | rs3821952 | INTRON 12 | 23406831 | 4.6 | 0.864 | 99.75 |
| PPARGC1A | rs4235308 | INTRON 2 | 23473510 | 40.5 | 0.397 | 99.75 |
| PPARGC1A | rs4361373 | INTRON 2 | 23484773 | 17.2 | 0.518 | 99.75 |
| PPARGC1A | rs4469064 | INTRON 2 | 23492894 | 10.0 | 0.555 | 100 |
| PPARGC1A | rs4568225 | PROMOTER | 23507528 | 28.0 | 0.036 | 99.75 |
| PPARGC1A | rs4697046 | INTRON 2 | 23445498 | 35.3 | 0.260 | 99.75 |
| PPARGC1A | rs6838835 | INTRON 2 | 23461430 | 0.6 | 0.900 | 99.75 |
| PPARGC1A | rs6850464 | INTRON 2 | 23468580 | 16.5 | 0.148 | 99.75 |
| PPARGC1A | rs725289 | INTRON 2 | 23487876 | 0.4 | 0.940 | 100 |
| PPARGC1A | rs7665116 | INTRON 2 | 23462109 | 13.6 | 0.795 | 99.75 |
| PPARGC1A | rs7672915 | INTRON 2 | 23465590 | 45.5 | 0.507 | 85.11 |
| PPARGC1A | rs7677000 | INTRON 2 | 23478552 | 18.8 | 0.172 | 99.75 |
| PPARGC1A | rs7682765 | INTRON 12 | 23412305 | 5.5 | 0.443 | 99.75 |
| PPARGC1A | rs768695 | INTRON 12 | 23407916 | 48.8 | 0.376 | 99.75 |
| HWE, Hardy-Weinberg equilibrium; MAF, minor allele frequency. | | | | | | |

| **Table S7B.Characteristics of 22 SNPs in the PPARA gene.** | | | | | | |
| --- | --- | --- | --- | --- | --- | --- |
| Gene | Marker (refSNP) | Gene Context | Chromosomal Position | MAF (%) | HWE P-value | Call rate (%) |
| PPARA | rs135543 | INTRON 2 | 44933985 | 27.5 | 0.490 | 99.26 |
| PPARA | rs1800206 | EXON 5 | 44992938 | 6.3 | 0.244 | 100 |
| PPARA | rs1800246 | INTRON 5 | 44994289 | 2.1 | 0.665 | 99.50 |
| PPARA | rs4253617 | INTRON 2 | 44927264 | 0.2 | 0.960 | 99.75 |
| PPARA | rs4253623 | INTRON 2 | 44928770 | 12.0 | 0.125 | 99.26 |
| PPARA | rs4253640 | INTRON 2 | 44942002 | 0.2 | <0.001 | 100 |
| PPARA | rs4253649 | INTRON 2 | 44943292 | 36.3 | 0.670 | 99.75 |
| PPARA | rs4253653 | INTRON 2 | 44947566 | 0.2 | 0.960 | 100 |
| PPARA | rs4253655 | INTRON 2 | 44947835 | 20.4 | 0.852 | 99.26 |
| PPARA | rs4253681 | INTRON 2 | 44958264 | 17.7 | 0.332 | 96.77 |
| PPARA | rs4253711 | INTRON 3 | 44973697 | 23.1 | 0.303 | 99.50 |
| PPARA | rs4253725 | INTRON 3 | 44987582 | 30.7 | 0.685 | 99.50 |
| PPARA | rs4253728 | INTRON 3 | 44988731 | 30.6 | 0.554 | 99.26 |
| PPARA | rs4253744 | INTRON 4 | 44991568 | 0.5 | 0.920 | 99.75 |
| PPARA | rs4253747 | INTRON 4 | 44991901 | 23.3 | 0.363 | 99.75 |
| PPARA | rs4253754 | INTRON 5 | 44993808 | 21.9 | 0.433 | 99.50 |
| PPARA | rs4253755 | INTRON 5 | 44994040 | 14.8 | 0.631 | 100 |
| PPARA | rs4253757 | INTRON 6 | 44994781 | 1.0 | <0.001 | 98.26 |
| PPARA | rs4253760 | INTRON 6 | 45001048 | 18.8 | 0.115 | 99.75 |
| PPARA | rs4253765 | INTRON 6 | 45001552 | 18.0 | 0.324 | 99.75 |
| PPARA | rs4253776 | INTRON 7 | 45008143 | 12.0 | 0.289 | 99.26 |
| PPARA | rs4253800 | 3' UTR | 45010115 | 0.9 | 0.860 | 99.75 |
| HWE, Hardy-Weinberg equilibrium; MAF, minor allele frequency. | | | | | | |

| **Table S7C. Characteristics of 17 SNPs in the PPARγ gene.** | | | | | | |
| --- | --- | --- | --- | --- | --- | --- |
| Gene | Marker (refSNP) | Gene Context | Chromosomal Position | MAF (%) | HWE P-value | Call rate (%) |
| PPARG | rs1151996 | INTRON 4 | 12420807 | 36.3 | 0.385 | 99.75 |
| PPARG | rs1151997 | INTRON 4 | 12421004 | 49.8 | 0.369 | 99.75 |
| PPARG | rs1175540 | INTRON 6 | 12440243 | 35.0 | 0.048 | 99.26 |
| PPARG | rs1175542 | INTRON 6 | 12441214 | 49.9 | 0.368 | 99.26 |
| PPARG | rs13073869 | INTRON 1 | 12328993 | 27.4 | 0.619 | 99.26 |
| PPARG | rs13095716 | INTRON 1 | 12328612 | 27.4 | 0.598 | 99.75 |
| PPARG | rs1373642 | INTRON 5 | 12424651 | 0.4 | 0.940 | 99.75 |
| PPARG | rs17247739 | INTRON 5 | 12422964 | 0.5 | 0.920 | 99.75 |
| PPARG | rs17247978 | INTRON 4 | 12414329 | 3.6 | 0.453 | 99.50 |
| PPARG | rs17425949 | INTRON 1 | 12331158 | 27.4 | 0.598 | 99.75 |
| PPARG | rs17435724 | INTRON 1 | 12377474 | 32.4 | 0.504 | 99.26 |
| PPARG | rs1797912 | INTRON 6 | 12445239 | 37.6 | 0.104 | 99.26 |
| PPARG | rs2959268 | INTRON 3 | 12405075 | 36.3 | 0.279 | 99.75 |
| PPARG | rs2959272 | INTRON 4 | 12417833 | 49.3 | 0.271 | 99.75 |
| PPARG | rs2972162 | INTRON 3 | 12399793 | 48.4 | 0.438 | 98.51 |
| PPARG | rs3856806 | EXON 7 | 12450557 | 14.6 | 0.874 | 97.77 |
| PPARG | rs4135247 | INTRON 1 | 12371588 | 41.0 | 0.166 | 99.75 |
| HWE, Hardy-Weinberg equilibrium; MAF, minor allele frequency. | | | | | | |

**Supplementary Figures**

**
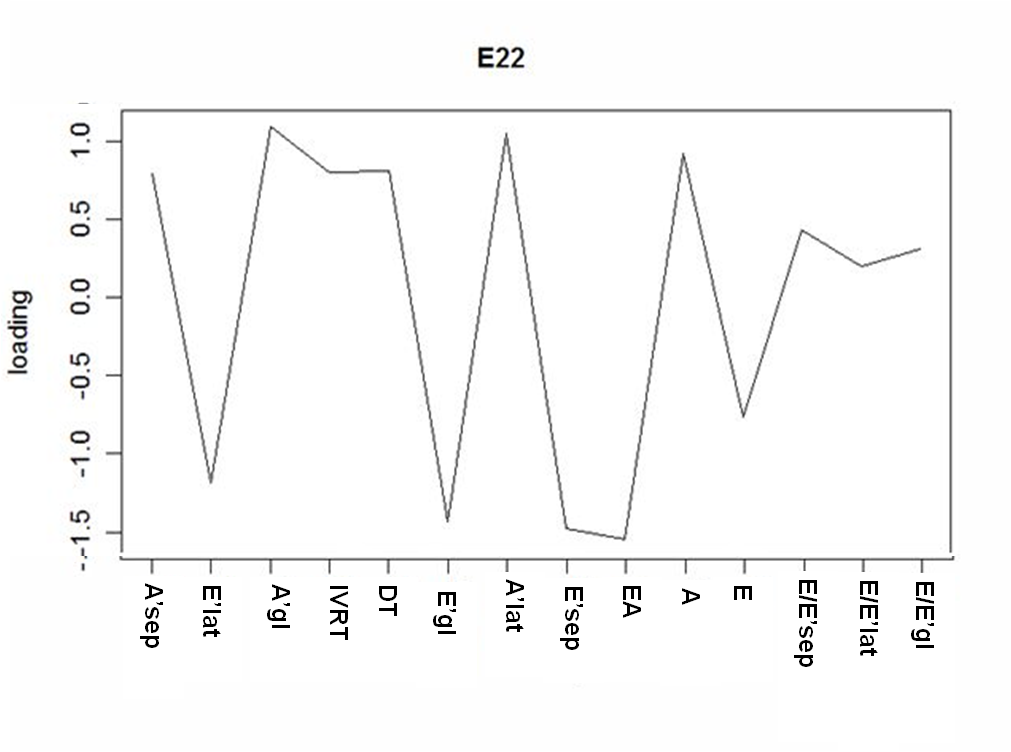
**


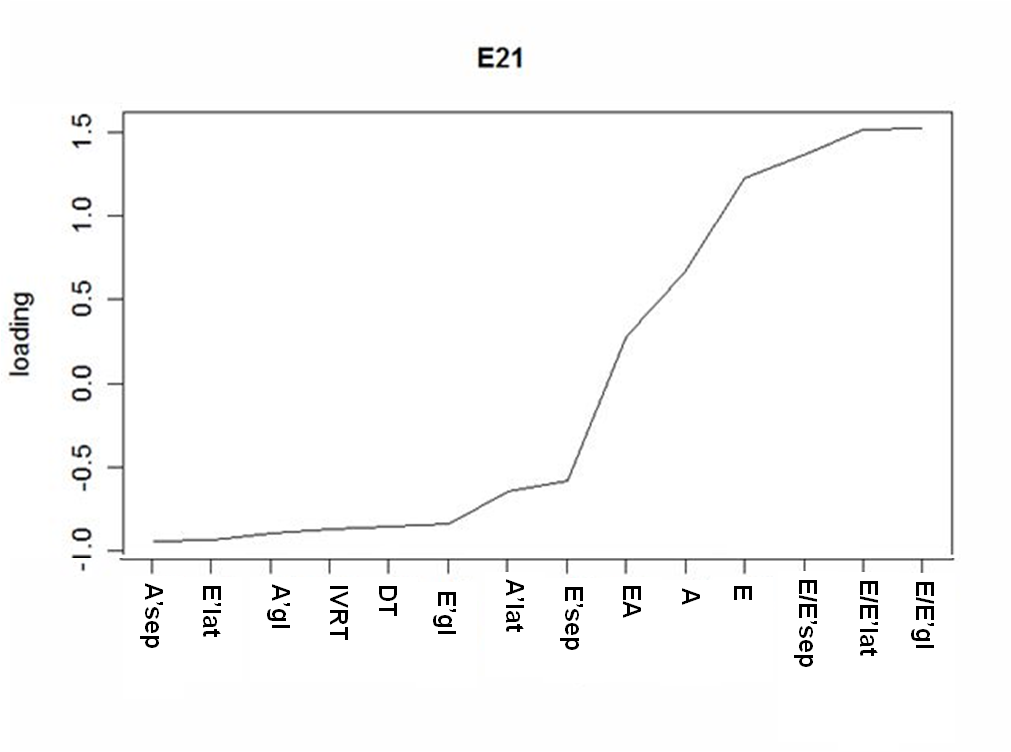


**Figure S1.** Plots of echocardiographic endophenotype loadings of two latent LVDD-related components, E21 (left) and E22 (right). In order to facilitate comparisons, the phenotypes on all graphs in S1-S3 were reordered from lowest to highest according to the loadings of E21 (chosen arbitrarily).


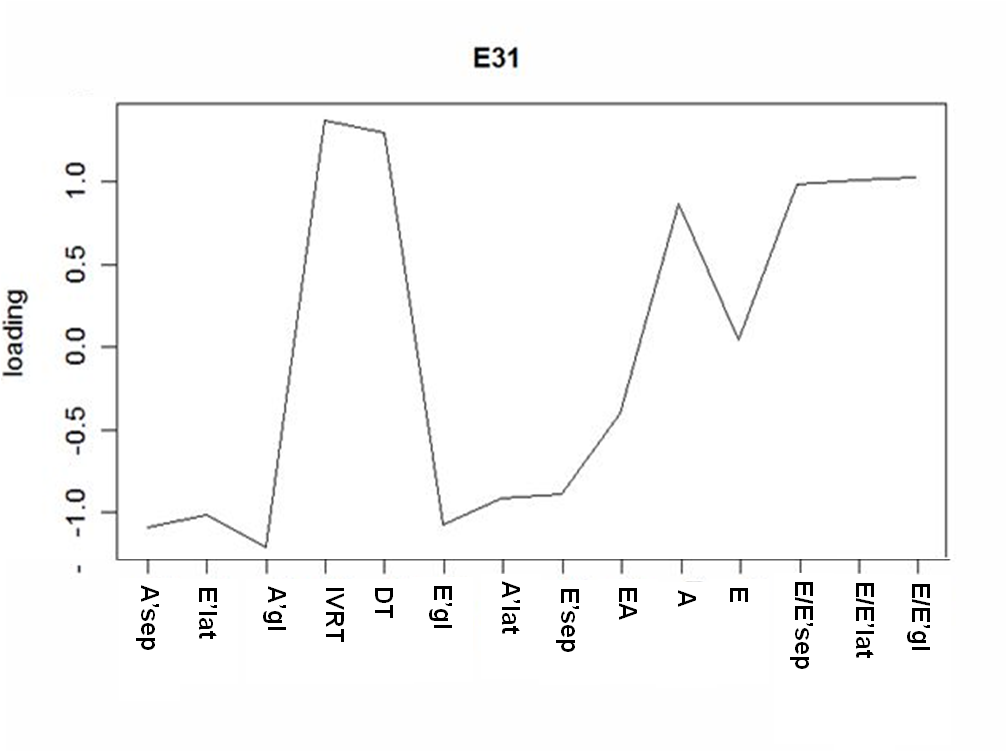

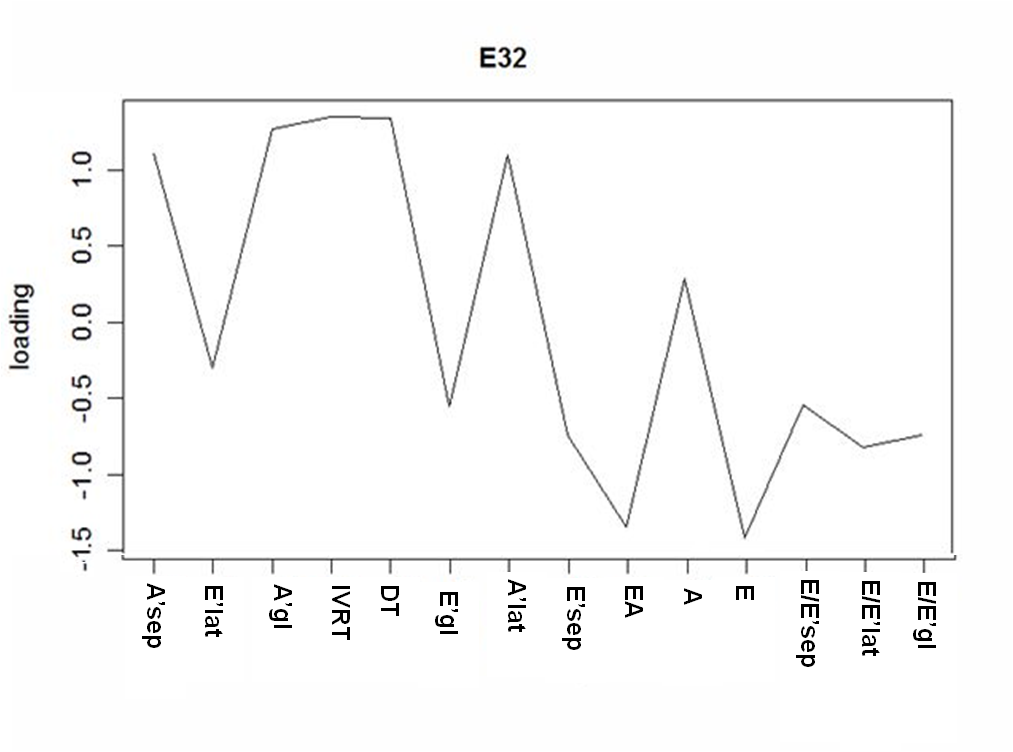

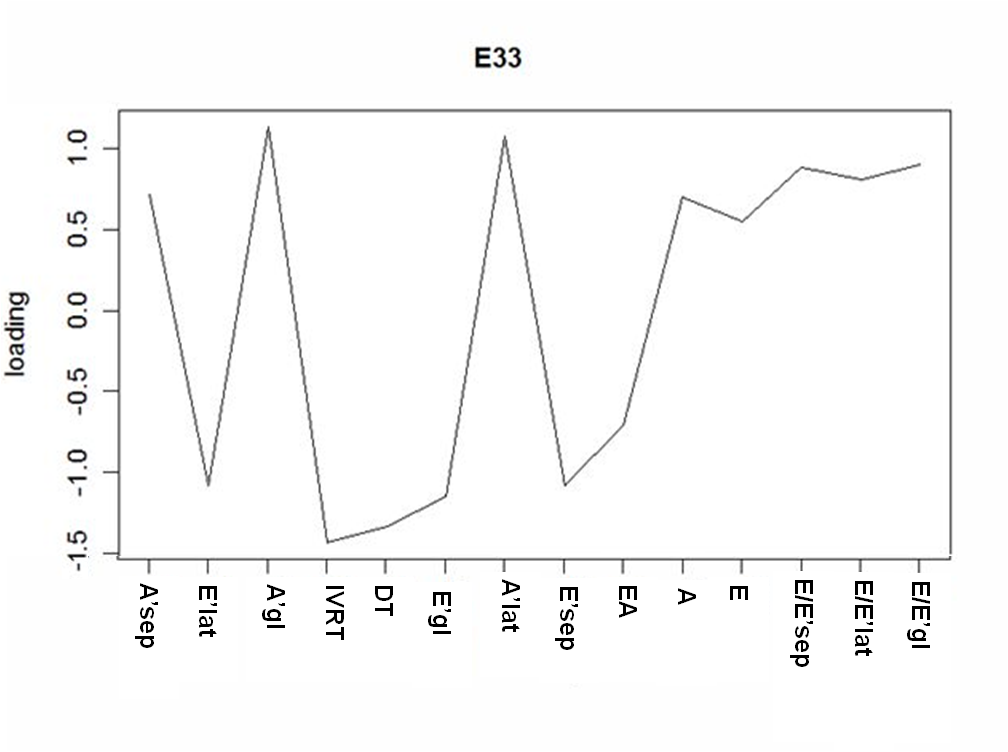


Figure S2. Plots of echocardiographic endophenotype loadings of E31, E32 and E33. In order to facilitate comparisons, the phenotypes on both graphs were reordered from lowest to highest according to the loadings of E21 (chosen arbitrarily).


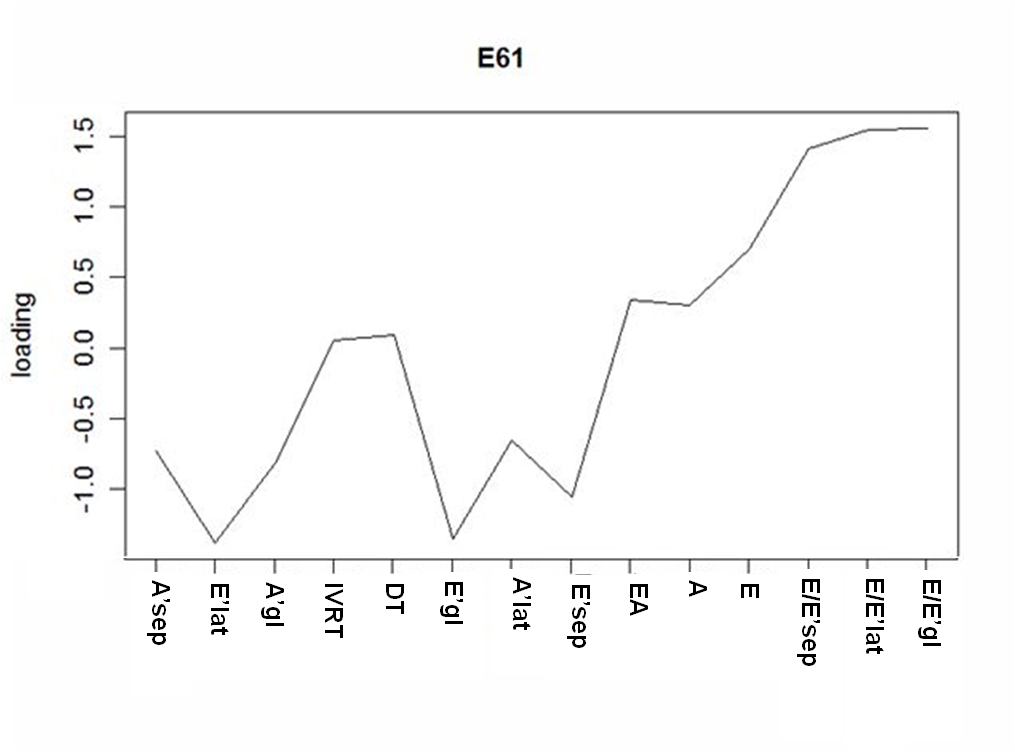

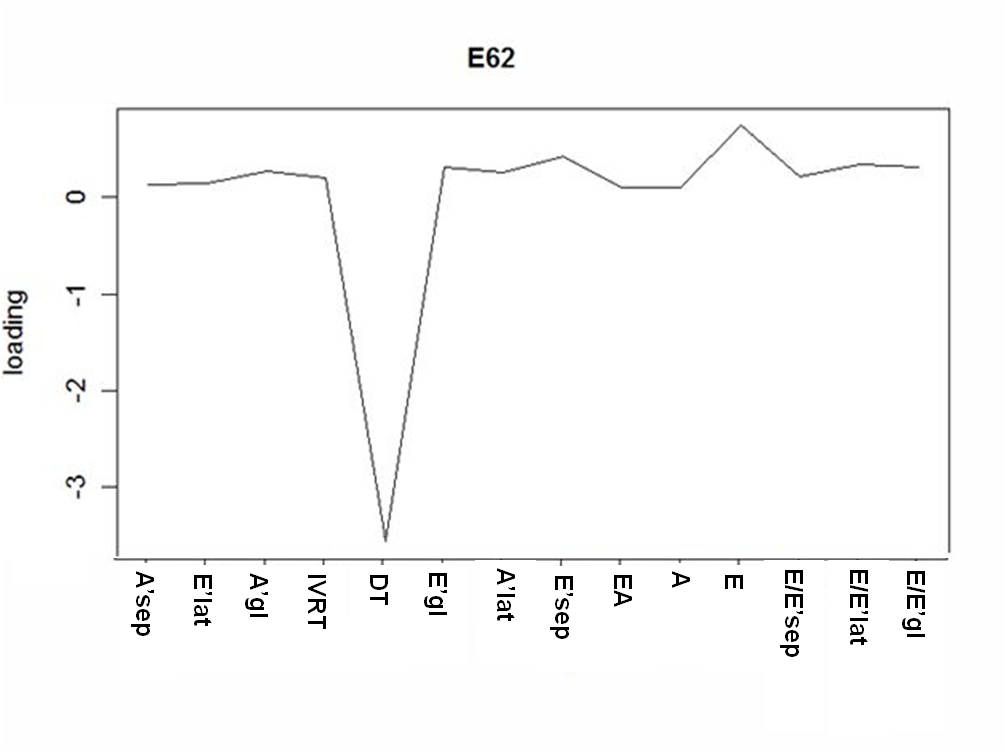

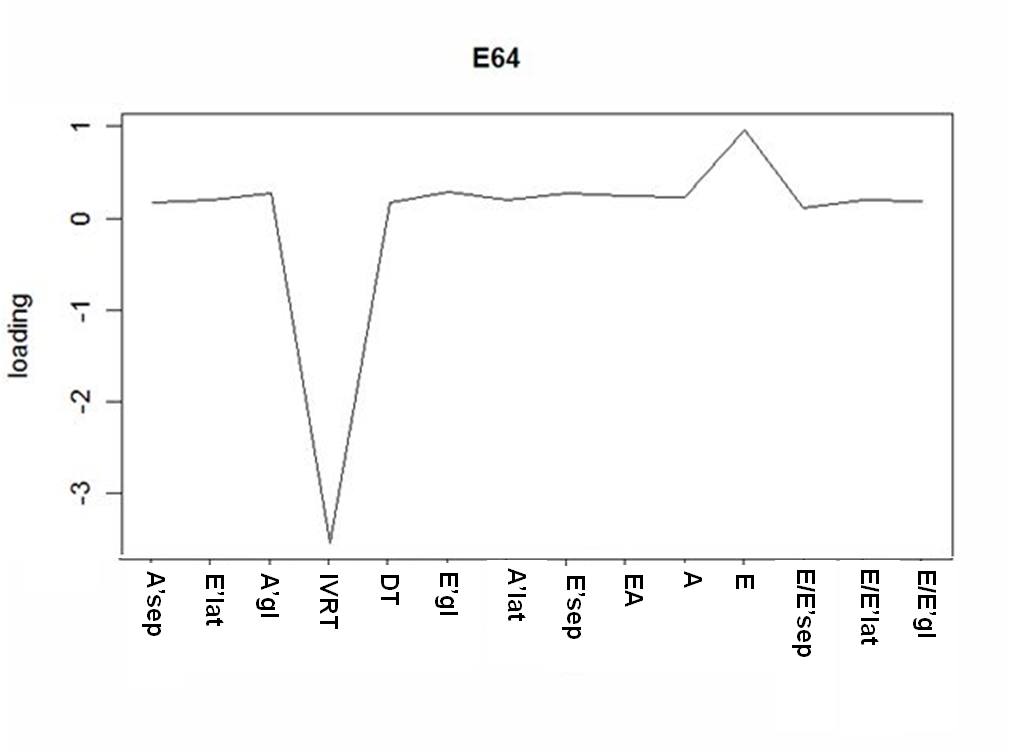

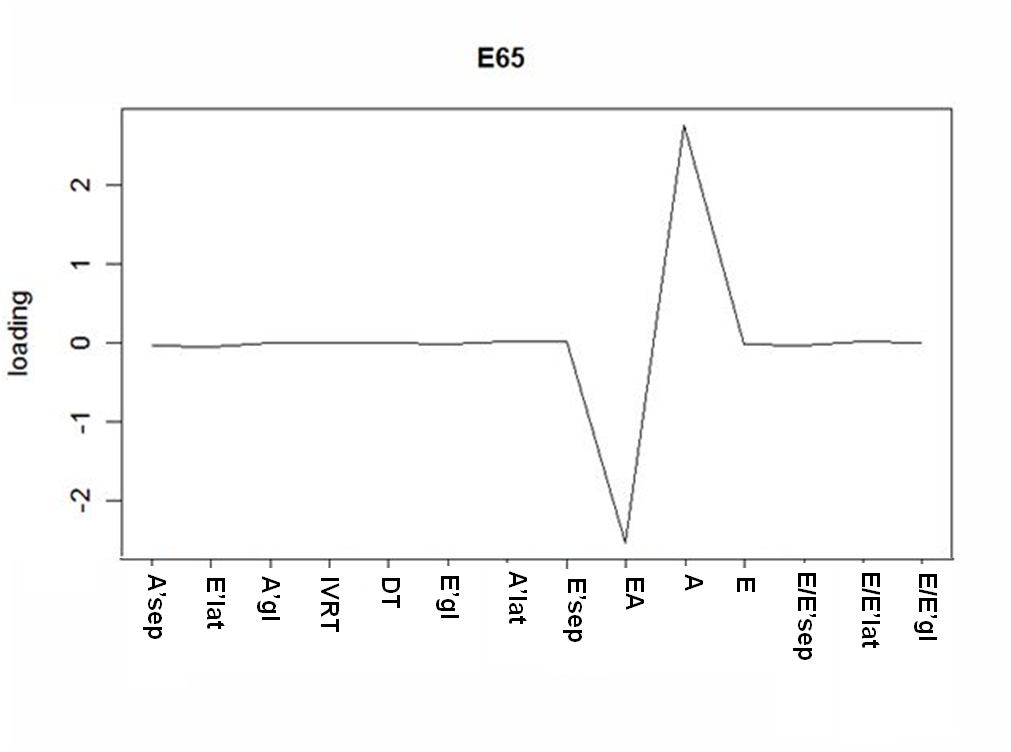

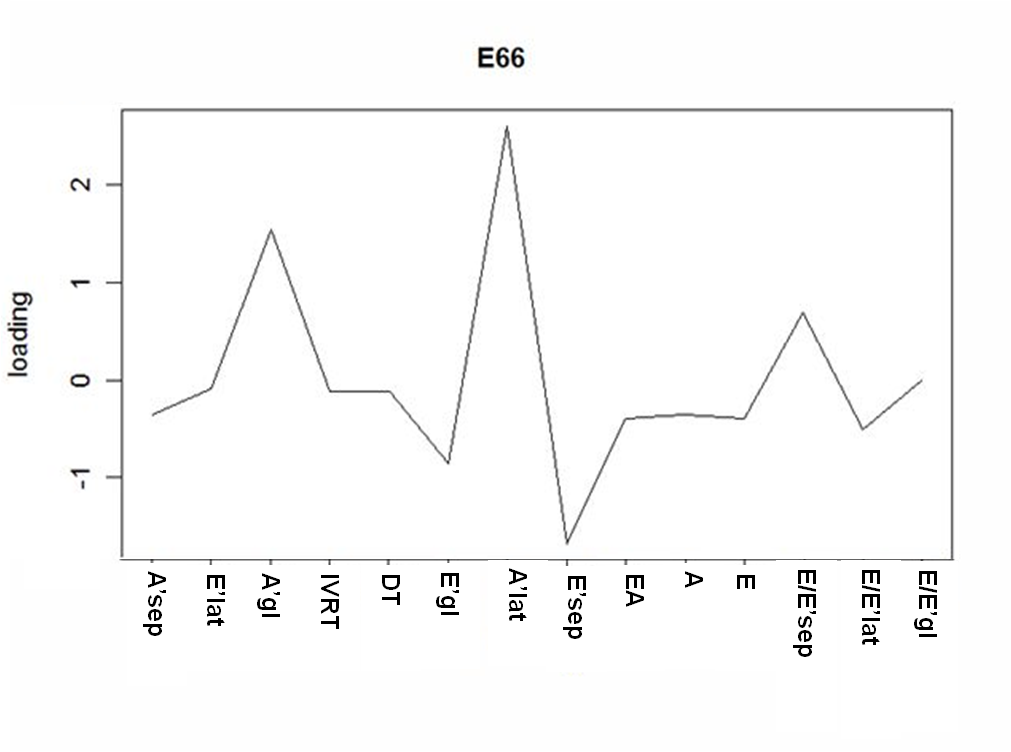


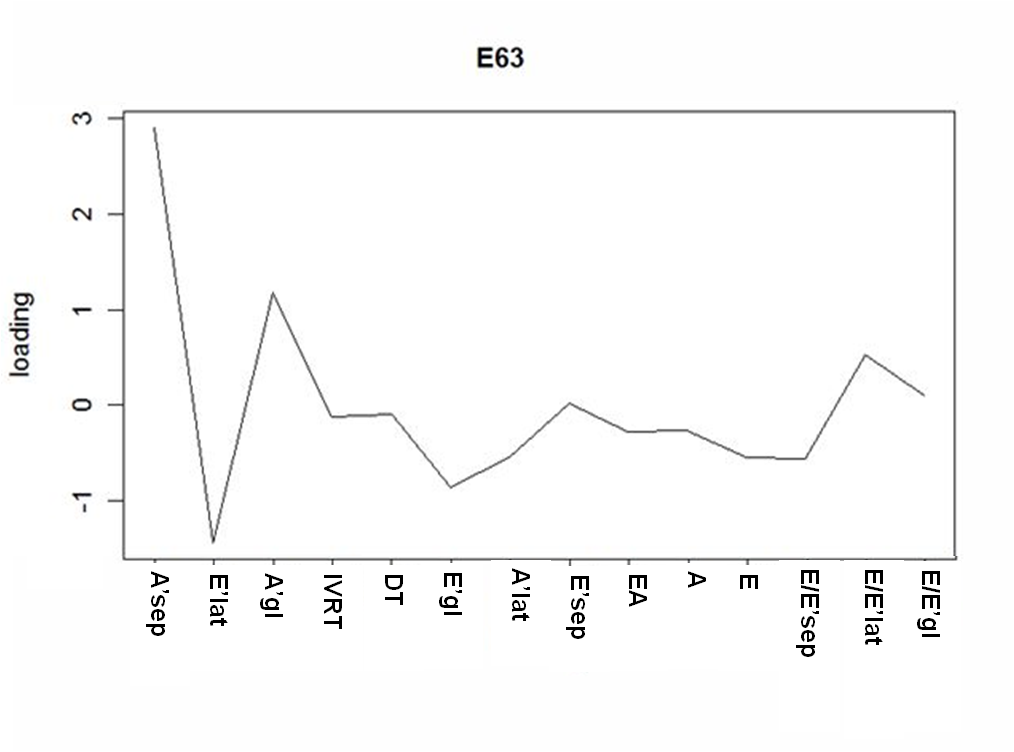
**Figure S3.** Plots of echocardiographic endophenotype loadings of E61-E66. In order to facilitate comparisons, the phenotypes on all graphs were reordered from lowest to highest according to the loadings of E21 (chosen arbitrarily).


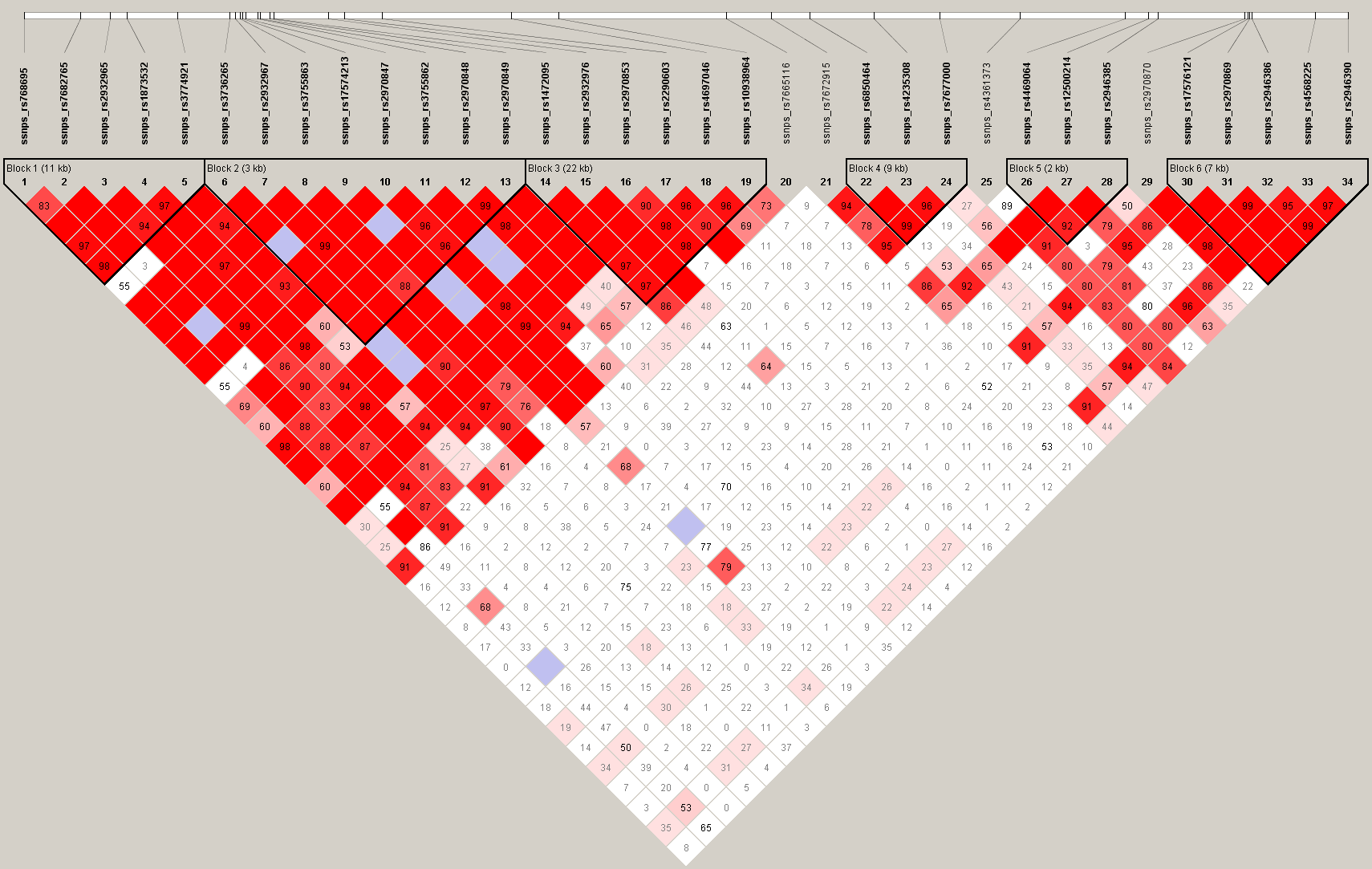


**Figure S4.** Haploview LD display of 34 PPARGC1A SNPs. This gene is transcribed off the reverse stand, so the promoter and 5’ end is on the right side of the figure.


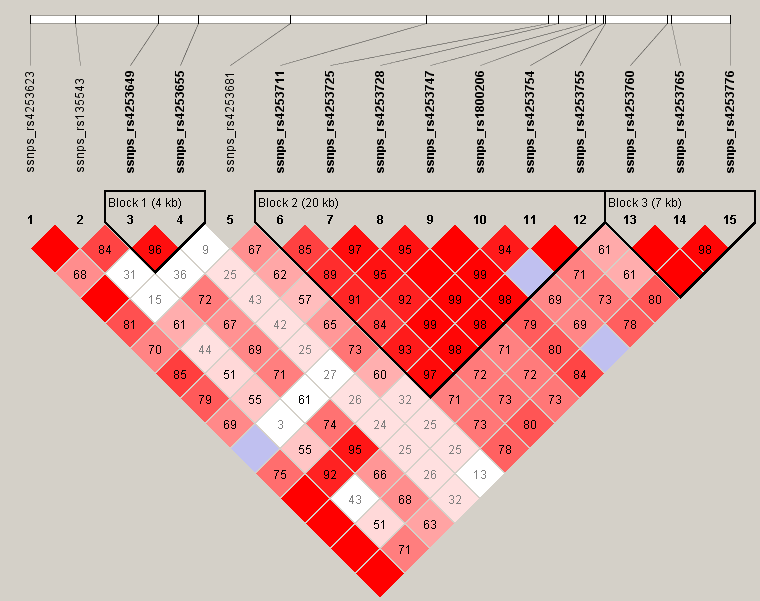


**Figure S5.** Haploview LD display of 15 PPARA SNPs. There were 3 significant SNPs in the block 3.


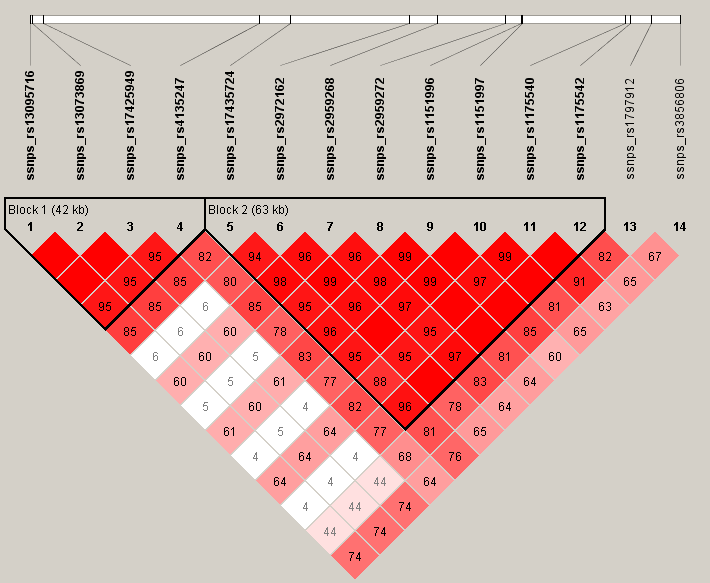


**Figure S6.** Haploview LD display of the 14 PPARG SNPs.


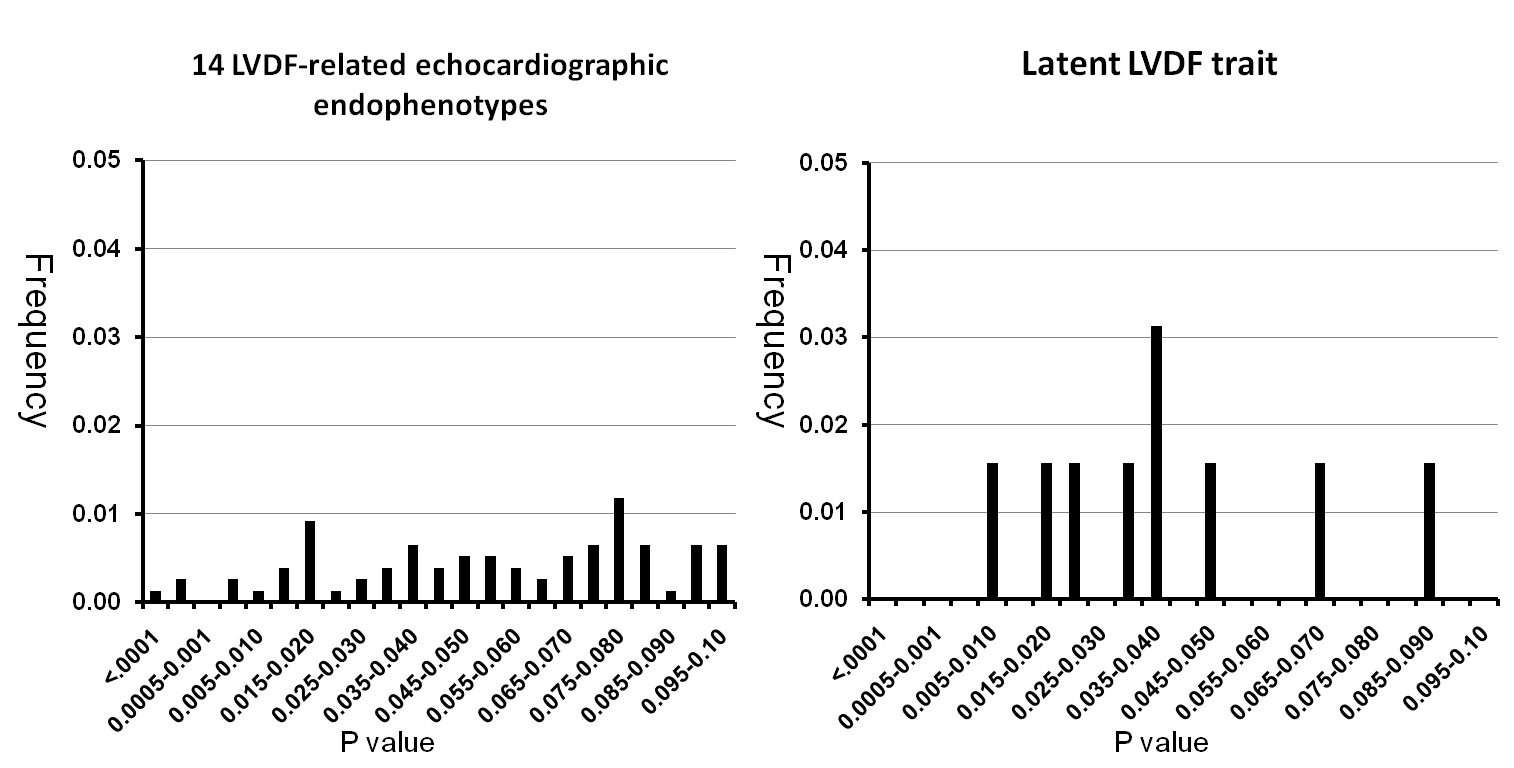


**Figure S7.** Comparison of the distributions of P values between latent LVDD trait (E61) and primary 14 echocardiographic endophenotypes.
